# Senataxin and DNA-PKcs redundantly promote non-homologous end joining repair of DNA double strand breaks during V(D)J recombination

**DOI:** 10.1126/sciadv.ads5272

**Published:** 2025-06-20

**Authors:** Bo-Ruei Chen, Thu Pham, Lance D. Reynolds, Nghi Dang, Yanfeng Zhang, Kimberly Manalang, Gabriel Matos-Rodrigues, Jason Romero Neidigk, Andre Nussenzweig, Jessica K. Tyler, Barry P. Sleckman

**Affiliations:** ^1^ Division of Hematology and Oncology, University of Alabama at Birmingham, Birmingham, AL 35233, USA.; ^2^ O’Neal Comprehensive Cancer Center, University of Alabama at Birmingham, Birmingham, AL 35233, USA.; ^3^ Genetics Research Division, University of Alabama at Birmingham, Birmingham, AL 35233, USA.; ^4^ Laboratory of Genome Integrity, National Cancer Institute, Bethesda, MD 20892, USA.; ^5^ Department of Pathology and Laboratory Medicine, Weill Cornell Medicine, New York, NY 10065, USA.

## Abstract

Nonhomologous end joining (NHEJ) is required for repairing DNA double strand breaks (DSBs) generated by the RAG endonuclease during lymphocyte antigen receptor gene assembly by V(D)J recombination. The ataxia telangiectasia–mutated (ATM) and DNA-dependent protein kinase catalytic subunit (DNA-PKcs) kinases regulate functionally redundant pathways required for NHEJ. Here, we report that loss of the senataxin helicase leads to a strong defect in RAG DSB repair upon inactivation of DNA-PKcs. The NHEJ function of senataxin is redundant with the RECQL5 helicase and the HLTF translocase and is epistatic with ATM. Co-inactivation of ATM, RECQL5, and HLTF results in an NHEJ defect similar to that from the combined deficiency of DNA-PKcs and senataxin or losing senataxin, RECQL5, and HLTF. These data suggest that ATM and DNA-PKcs regulate the functions of senataxin and RECQL5/HLTF, respectively, to provide redundant support for NHEJ.

## Teaser

Genetic pathways mediated by helicases and kinases redundantly promote DNA DSB repair.

## INTRODUCTION

The genes that encode antigen receptors are assembled in developing lymphocytes through the process of V(D)J recombination. V(D)J recombination is initiated when the recombination-activating gene 1 (RAG1) and RAG2 proteins, which together form the RAG endonuclease, introduce DNA double-strand breaks (DSBs) at the borders of a pair of recombining variable (V), diversity (D), or joining (J) gene segments and their adjacent RAG recognition sequences termed recombination signal sequences (RSSs) (*1*, *2*). This reaction leads to the formation of a blunt signal end and a hairpin-sealed coding end at each RAG DSB. These DNA ends are then processed and repaired by the nonhomologous end joining (NHEJ) pathway of DNA DSB repair. The two blunt signal ends (SEs) are directly ligated to generate a signal joint (SJ), while the two hairpin-sealed coding ends (CEs) are first opened by Artemis before ligation to form a coding joint (CJ) (*3*, *4*). Signal ends join precisely with occasional loss of a few nucleotides, whereas coding ends are joined imprecisely with most joints undergoing nucleotide gain or loss (*3*, *4*).

The NHEJ pathway of DSB repair functions in all phases of the cell cycle, with the exception of mitosis, and relies on the core factors KU70, KU80, DNA ligase IV (LIG4) and XRCC4, which are all essential for NHEJ-mediated DSB repair (*5*–*7*). Loss of any of these factors leads to a complete block in NHEJ-mediated DSB repair. Additional NHEJ factors may be required for the repair of DSBs with unique end structures, such as Artemis, a nuclease that is needed to open the hairpin-sealed coding ends during V(D)J recombination (*8*). Recent studies using cell biological, biochemical, structural, and single molecular approaches have greatly advanced the understanding of the molecular mechanisms of NHEJ (*9*, *10*). DSBs to be repaired by NHEJ are recognized and quickly bound by the KU70/KU80 heterodimer, which subsequently recruits and activates DNA-dependent protein kinase catalytic subunit (DNA-PKcs). In addition to its well-known function as a serine/threonine kinase in DNA damage response (DDR) via phosphorylating other proteins, DNA-PKcs has a structural role in establishing the long-range synaptic complex that serves to stabilize and protect DNA ends. Upon DNA-PKcs autophosphorylation, the DNA ends become available for short-range synapsis mediated by NHEJ factors including LIG4, XRCC4, and XLF, leading the ligation of broken DNA strands (*11*–*16*). In addition to the aforementioned core factors, accessory NHEJ proteins including XRCC4-Like Factor (XLF), Paralog Of XRCC4 And XLF (PAXX), and Modulator Of Retrovirus Infection (MRI) and DDR factors such as H2AX and 53BP1 have also been shown to redundantly promote efficient NHEJ with the individual loss of any of these proteins leading to a modest defect in NHEJ, while the combined inactivation of some of them severely impedes the repair process (*17*).

The serine/threonine kinases ataxia telangiectasia mutated (ATM) and DNA-PKcs are activated early in the response to DNA DSBs to regulate pathways required for NHEJ through phosphorylation of many downstream effectors (*18*). DNA-PKcs is activated by DSBs upon recruitment to broken DNA ends by the KU70/KU80 heterodimer, while ATM activation in response to DSBs is mediated by the MRE11/RAD50/NBS1 (MRN) complex (*18*). Although ATM and DNA-PKcs are known to mediate many important processes during the DNA damage response and repair, loss of either ATM or DNA-PKcs only leads to limited defects in NHEJ. In contrast, the combined loss of function of both kinases results in a stronger defect in NHEJ, demonstrating that they control key NHEJ repair activities that function redundantly (*19*–*22*).

To reveal novel factors that are regulated by ATM or DNA-PKcs kinases and function redundantly during NHEJ, we carried out CRISPR-Cas9 whole-genome guide RNA (gRNA) screens in Abelson murine leukemia viral kinase-transformed precursor B (pre-B) cell lines, hereafter referred to as abl pre-B cells. Treatment of abl pre-B cells with the abl kinase inhibitor imatinib leads to cell cycle arrest in G_0_ phase and initiation of V(D)J recombination (*23*–*25*). Intrigued by the well-demonstrated roles of DNA-PKcs during NHEJ in recent structural and single-molecular studies and the paradoxical lack of effect on the repair of RAG-generated DSBs in DNA-PKcs–inhibited cells, we first sought to uncover factors that may function in parallel with DNA-PKcs to promote NHEJ by CRSPR-Cas9 screens on abl pre-B cells with or without DNA-PKcs inhibition. We found that gRNAs to the senataxin gene (*Setx*) strongly impeded NHEJ-mediated repair of RAG DSBs in G_0_-arrested abl pre-B cells treated the DNA-PKcs inhibitor NU7441, but not in the untreated cells. Senataxin is a member of the superfamily 1 (SF1) RNA helicases that unwinds RNA/DNA hybrids, including R loops, a three-strand structure composed of an RNA/DNA hybrid and displaced nontemplate single-strand DNA (ssDNA) (*26*–*28*). Here, we established that senataxin is required for NHEJ in cells where DNA-PKcs is inactivated and that senataxin acts redundantly with the RECQL5 helicase and the Helicase-Like Transcription Factor (HLTF) translocase. This is the first evidence implicating multiple collaborating helicase and translocase activities in NHEJ in ATM- and DNA-PKcs–dependent manner.

## RESULTS

### Senataxin is required for RAG DSB repair in DNA-PKcs–deficient abl pre-B cells

**Fig. 1.**
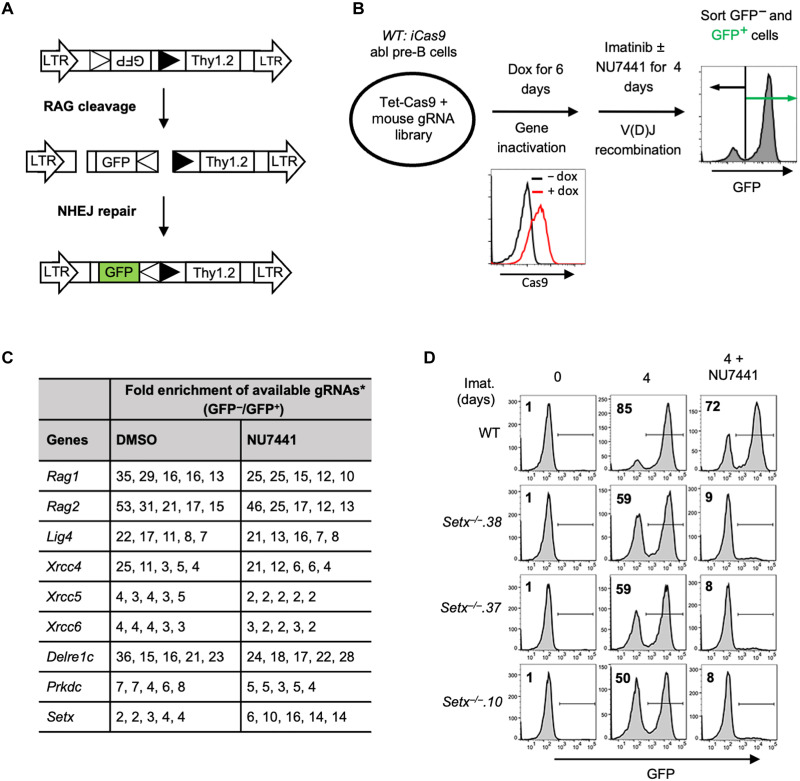
A Genome-wide CRISPR/Cas9 screen for identifying genes required for V(D)J recombination in DNA-PKcs inhibited abl pre-B cells. (**A**) Schematic of the retroviral V(D)J recombination substrate pMG-INV. The open and filled triangles represent the RSSs. The retroviral LTRs, CEs, and SEs upon RAG cleavage and CJs and SJs after NHEJ-mediated repair are indicated. The anti-sense GFP cDNA is inverted to the sense orientation upon completion of the recombination and its expression driven by the retroviral LTR (green). (**B**) Schematic diagram of a genome-wide CRISPR-Cas9 screen in WT abl pre-B cells for identifying genes important for V(D)J recombination upon DNA-PKcs inhibition with NU7441. (**C**) Fold enrichments of selected gRNAs from screens conducted in DMSO and NU7441-treated WT abl pre-B cells (both also treated with imatinib). The fold enrichment is calculated as the ratio of the normalized read counts of a gRNA from GFP^−^ cells over that from GFP^+^ cells. Asterisk denotes (*) five gRNAs to each gene. (**D**) Flow cytometric analysis for GFP expression in WT and three independently isolated *Setx*^−/−^ abl pre-B cell lines with pMG-INV and treated with imatinib (imat.) in the presence or absence of the DNA-PKcs kinase inhibitor NU7441 for the indicated times. The percentages of GFP^+^ cells are indicated in the top left corners of the histograms.

V(D)J recombination in abl pre-B cells can be assayed with chromosomally integrated retroviral recombination substrates that contain a single pair of RSSs (*24*, *29*). The pMG-INV recombination substrate contains an anti-sense green fluorescent protein (GFP) cDNA flanked by \RSSs oriented such that successful V(D)J recombination leads to inversion of the GFP cDNA and GFP expression (Fig. 1A) (*29*). To identify genes critical for NHEJ-mediated RAG DSB repair in cells lacking DNA-PKcs kinase activity, wild-type (WT) abl pre-B cells containing a single copy of the pMG-INV recombination substrate were transduced with a whole-genome lentiviral gRNA library and treated with imatinib to induce V(D)J recombination in the presence of the DNA-PKcs inhibitor NU7441 or vehicle [dimethyl sulfoxide (DMSO)] alone. Cells that completed V(D)J recombination (GFP^+^) and those that did not (GFP^−^) were isolated by fluorescence-activated cell sorting (FACS), and the gRNAs in these cells were amplified by PCR and sequenced (Fig. 1B). Among the gRNAs highly enriched in GFP^−^ populations in both vehicle- and NU7441-treated abl pre-B cells were those to the genes encoding NHEJ factors (e.g *Lig4*, *Xrcc4*, *Xrcc5*, *Xrcc6, Delre1c and Prkdc*) and genes required for generating RAG DSBs (e.g. *Rag1* and *Rag2*), thus validating our screening approach (Fig. 1C and table S1). The gRNAs to *Setx*, a gene which encodes the protein senataxin, were three- to fivefold more enriched in the GFP^−^ population of abl pre-B cells treated with NU7441 as compared to vehicle alone, suggesting that senataxin is required for V(D)J recombination in abl pre-B cells where DNA-PKcs kinase activity has been inhibited (Fig. 1C).

*Setx*^−/−^ abl pre-B cell clones were generated by CRISPR-Cas9–mediated gene editing, and the inactivating mutations were verified by sequencing (table S2). Flow cytometric analyses of GFP expression revealed that in comparison to WT abl pre-B cells, *Setx*^−/−^ abl pre-B cells with integrated pMG-INV exhibited a mild reduction in V(D)J recombination efficiency as evidenced by the slightly diminished percentage of GFP^+^ cells after imatinib treatment. However, treatment with the DNA-PKcs kinase inhibitor NU7441 led to a notable reduction in GFP^+^ *Setx*^−/−^ abl pre-B cells as compared to WT abl pre-B cells (Fig. 1D and table S3A). We conclude that senataxin is required for efficient V(D)J recombination in abl pre-B cells that have compromised DNA-PKcs kinase activity.

**Fig. 2.**
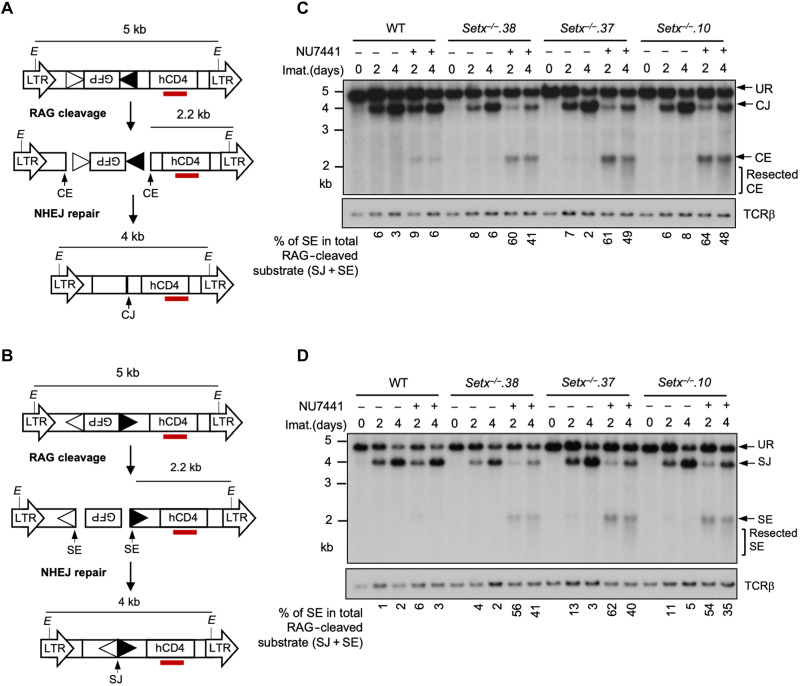
Loss of senataxin severely impairs NHEJ-mediated RAG DSB repair in DNA-PKcs-inhibited abl pre-B cells during V(D)J recombination. (**A** and **B**) Schematic of the retroviral V(D)J recombination substrates pMX-DEL^CJ^ (A) and pMX-DEL^SJ^ (B). The open and filled triangles represent the RSSs. The red bar indicates the probe (hCD4 cDNA) for Southern blot. The Es denote the Eco RV restriction sequences. The retroviral LTRs, CEs, and SEs upon RAG cleavage and CJs and SJs after NHEJ-mediated repair are indicated. (**C** and **D**) Southern blot analysis of genomic DNA from WT and three clonal *Setx*^−/−^ abl pre-B cell lines with pMX-DEL^CJ^ (C) or pMX-DEL^SJ^ (D) treated with imatinib in the presence or absence of the DNA-PKcs kinase inhibitor NU7441 for the indicated times. The genomic DNA samples were digested with Eco RV and hybridized with the hCD4 probe. The restriction fragments corresponding to unrearranged reporters (UR), repaired CJs or SJs, and unrepaired CEs or SEs are indicated. For all Southern blot analysis of pMX-DEL^SJ^ or pMX-DEL^CJ^, the percentages of unrepaired (full length and resected) SEs in total RAG cleaved recombination substrates (SE + SJ) are shown below the Southern blot images. β T cell receptor (TCRβ): loading control.

A deficiency in V(D)J recombination could result from defected RAG cleavage and/or impaired NHEJ-mediated repair of RAG DSBs. To determine whether senataxin is required for the repair of RAG DSBs during V(D)J recombination, we analyzed the recombination products of the pMG-INV substrate in imatinib-treated WT and *Setx*^−/−^ abl pre-B cells in the presence or absence of NU7441 by Southern blot (fig. S1, A and B). In both WT and *Setx*^−/−^ abl pre-B cells, treatment with imatinib alone did not lead to accumulation of unrepaired SEs (fig. S1B, Xba I digestion). Although *Setx*^−/−^ abl pre-B cells generated abundant repaired SJs, when compared to WT abl pre-B cells, these cells accumulated slightly higher levels of unrearranged (UR) substrate and unproductive recombination product hybrid joints (HJs), resulting from aberrant joining of a SE to a CE, likely accounting for the modest reduction of GFP-expressing in the flow cytometric assay (Fig. 1D and fig. S1, A and B). Upon DNA-PKcs inhibition by NU7441 treatment, SJ formation greatly diminished in *Setx*^−/−^ abl pre-B cells, accompanied by greater accumulation of the aberrant HJs and unrepaired SEs (fig. S1B). Therefore, defected and aberrant NHEJ play a major role in the V(D)J recombination defect observed in DNA-PKcs inhibited *Setx*^−/−^ abl pre-B cells. To further strengthen the RAG DSB repair defect in abl pre-B cells lacking senataxin and DNA-PKcs activity, we conducted Southern blot analysis on the pMX-DEL^CJ^ or pMX-DEL^SJ^ retroviral recombination substrates that allow for visualizing the repair of structurally different CEs and SEs and the formation of CJs and SJs, respectively (Fig. 2, A and B). The recombination of pMX-DEL^CJ^ and pMX-DEL^SJ^, which have RSSs positioned in opposite orientations, is accompanied by loss of the GFP cDNA bordered by RSSs. This creates either two chromosomal hairpin-sealed CEs (pMX-DEL^CJ^) or open, blunt SEs (pMX-DEL^SJ^) to be joined by NHEJ to form CJs or SJs (Fig. 2, A and B). Southern blot analysis of *Setx*^−/−^ abl pre-B cells containing pMX-DEL^CJ^ or pMX-DEL^SJ^ treated with imatinib and NU7441 revealed a marked reduction in the formation of CJs and SJs and increased accumulation of unrepaired CEs and SEs, respectively, as compared to imatinib treatment alone (Fig. 2, C and D, and figs. S2, A and B). In contrast, treatment of WT abl pre-B cells containing pMX-DEL^CJ^ or pMX-DEL^SJ^ with imatinib and NU7441 had minimal effects on CJ and SJ formation (Fig. 2, C and D, and fig. S2, A and B). These results indicate that while individual loss of senataxin has modest impact on inversional V(D)J recombination, as measured with the pMG-INV substrate, due to the formation of nonproductive HJs, *Setx*^*−*/−^ abl pre-B cells do not have a demonstrable defect RAG DSB repair. On the other hand, inactivating senataxin in abl pre-B cells lacking DNA-PKcs activity leads to a NHEJ defect during V(D)J recombination.

**Fig. 3.**
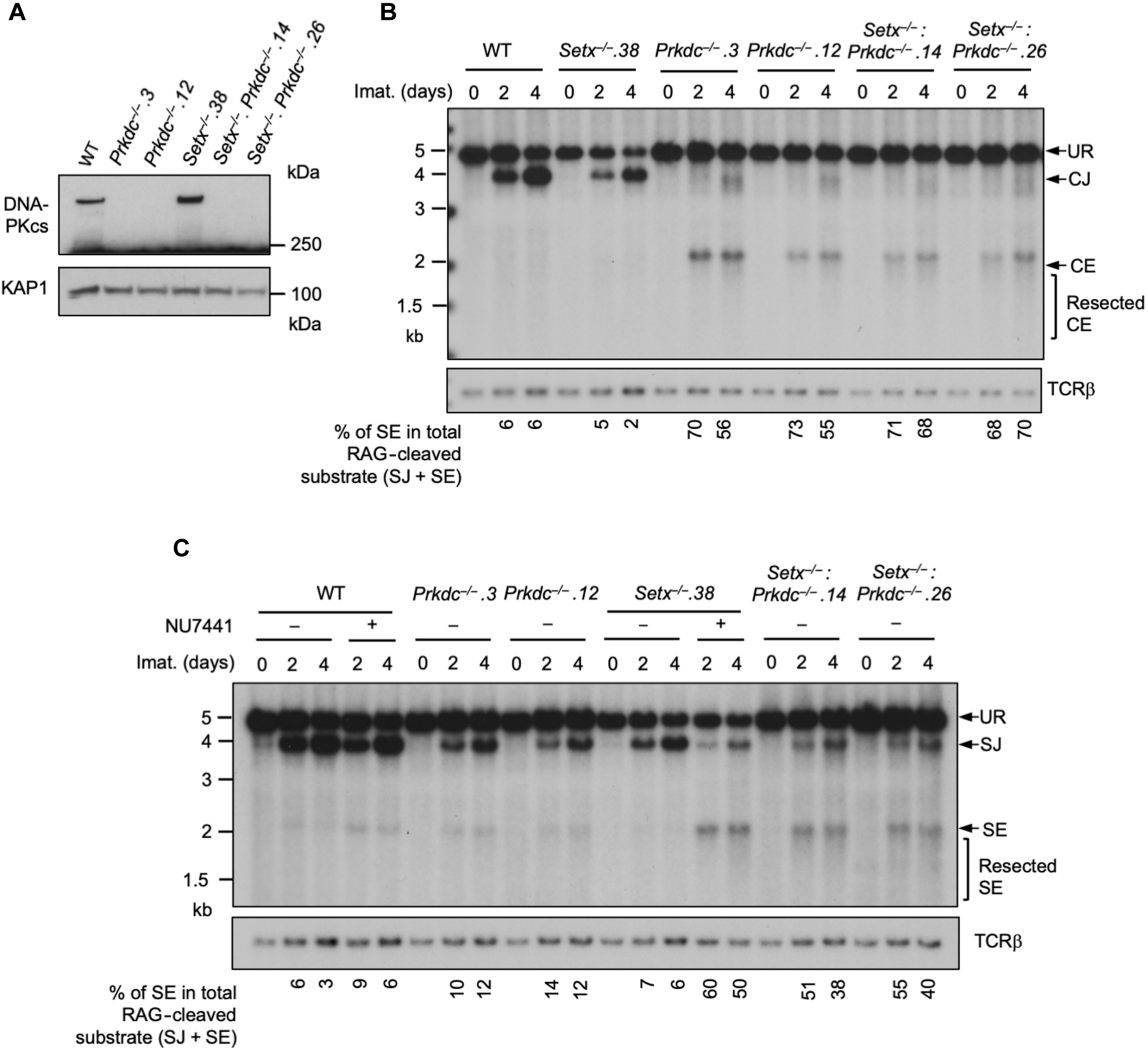
Combined loss of senataxin and DNA-PKcs proteins impairs NHEJ-mediated RAG DSB repair. (**A**) Western blot analysis of cell lysates from WT, *Prkdc*^−/−^, *Setx*^−/−^, and *Setx*^−/−^: *Prkdc*^−/−^ abl pre-B cells using DNA-PKcs and KAP1 antibodies. (**B** and **C**) Southern blot analysis of Eco RV–digested genomic DNA isolated from WT, *Prkdc*^−/−^, *Setx*^−/−^, and *Setx*^−/−^: *Prkdc*^−/−^ abl pre-B cells with pMX-DE^CJ^ (B) or pMX-DE^SJ^ (C) and treated with imatinib in the presence or absence of the DNA-PKcs inhibitor NU7441 for the indicated times.

We also generated *Setx*^*−*/−^: *Prkdc^−/−^* abl pre-B cells that lack both senataxin and DNA-PKcs proteins by inactivating *Prkdc*, which encodes DNA-PKcs, in *Setx*^−/−^ abl pre-B cells using CRISPR-Cas9 gene editing (Fig. 3A and fig. S3A). Although DNA-PKcs kinase activity is not required for opening the hairpin-sealed CEs by the nuclease ARTEMIS, DNA-PKcs protein is essential for this process. Therefore, CEs remained hairpin-sealed and CJs could not be formed in *Prkdc* null cells during V(D)J recombination regardless the status of senataxin (Fig. 3B) (*8*, *22*, *30*). However, in contrast to WT and *Prkdc^−/−^* abl pre-B cells where SJs were formed efficiently with only a little or no detectable SEs left unrepaired, *Setx*^*−*/−^: *Prkdc^−/−^* abl pre-B cells accumulated abundant unrepaired SEs after induction of V(D)J recombination (Fig. 3C and fig. S3B). Kinase-inactive DNA-PKcs or DNA-PKcs with autophosphorylated residues mutated have been shown to impede NHEJ by prolonged binding DNA ends and making DSBs inaccessible to repair factors (*30*–*32*). Our results that chemical inhibition or genetic ablation of DNA-PKcs in *Setx*^*−*/−^ abl pre-B cells resulted in similar repair defect suggest that DNA-PKcs inhibitor unlikely cause NHEJ deficiency in *Setx*^*−*/−^ abl pre-B cells by trapping DNA-PKcs at DNA ends. Note that the level of ATM protein was not altered in cells lacking senataxin and DNA-PKcs, indicating that the observed NHEJ defect is not due to loss of ATM and DNA-PKcs activities in *Setx*^−/−^: *Prkdc*^−/−^ abl pre-B cells (fig. S3C). We conclude that while individual loss of senataxin or DNA-PKcs kinase activity has minimal effects on RAG DSB repair, the combined loss of senataxin and DNA-PKcs kinase activity leads to a strong block in signal and coding end joining during V(D)J recombination.

**Fig. 4.**
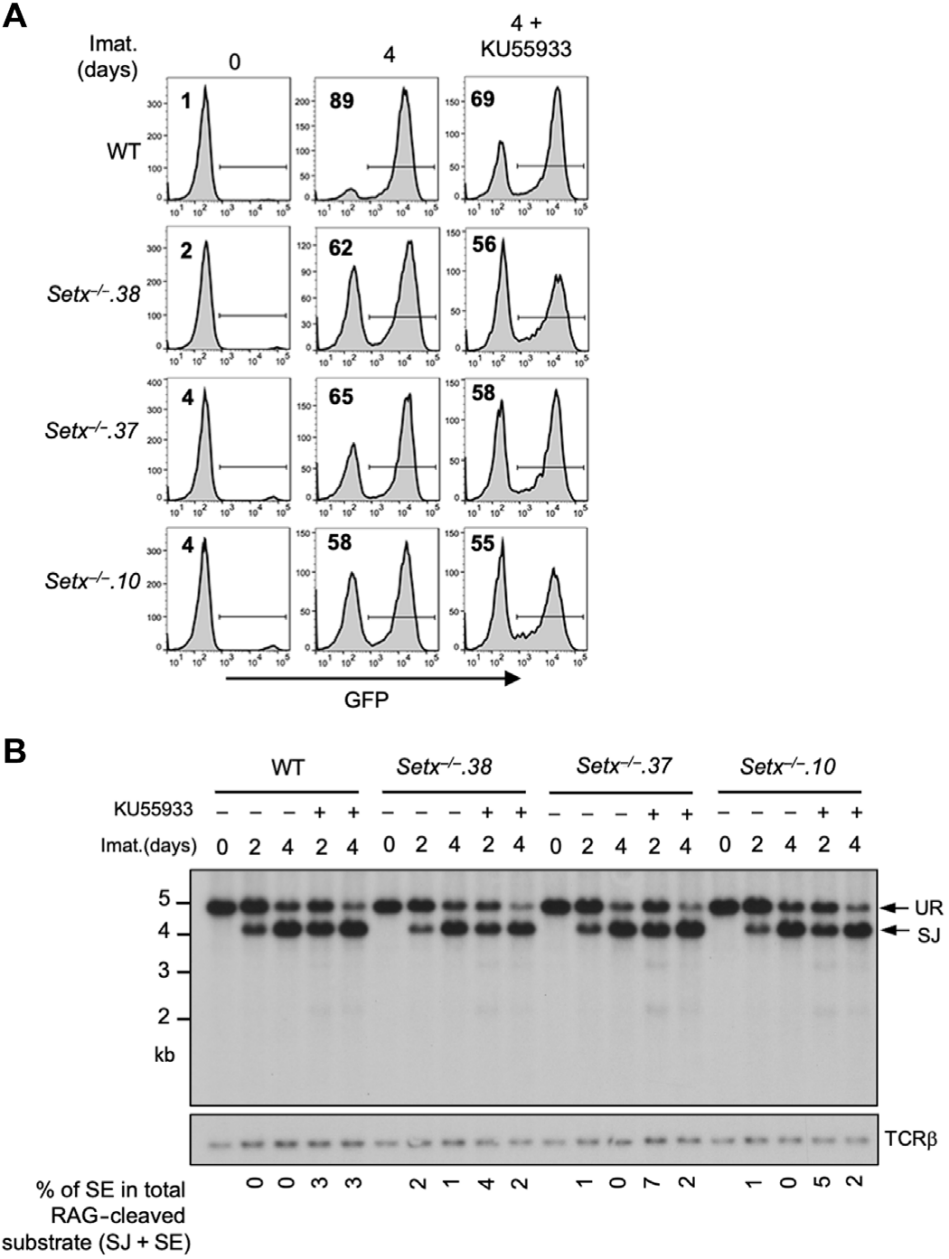
ATM inhibition in *Setx*^−/−^ abl pre-B cells does not affect V(D)J recombination. (**A**) Flow cytometric analysis for GFP expression in WT and *Setx*^−/−^ abl pre-B cell lines with pMG-INV and treated with imatinib in the presence or absence of the ATM kinase inhibitor KU55933 for the indicated times. The percentages of GFP^+^ cells are indicated in the top left corners of the histograms. (**B**) Southern blot analysis of Eco RV–digested genomic DNA isolated from WT and *Setx*^−/−^ abl pre-B cell lines with pMX-DE^SJ^ and treated with imatinib in the presence or absence of KU55933 for the indicated times.

In contrast to loss of DNA-PKcs activity, inhibition of ATM kinase activity by treating *Setx*^−/−^ abl pre-B cells with KU55933 had only small effect on V(D)J recombination as revealed by similar abundance of GFP-expressing cells in flow cytometric analysis of the pMG-INV substrate (Fig. 4A and table S3B). On the contrary, ATM inhibition in WT cells led to a profound loss of GFP^+^ cells due to the accumulation of nonproductive HJs during inversional recombination (Fig. 4A and fig. S1C) (*24*). While combined deficiencies in DNA-PKcs activity and senataxin resulted in higher levels of nonproductive HJs during inversional recombination of pMG-INV than in individual deficiencies (fig. S1B), the abundance of HJs remained similar in cells lacking ATM activity and senataxin individually or in combination (fig. S1C) (*24*). Last, Southern blot analysis of pMX-DEL^SJ^ demonstrated comparable levels of SJs and the lack of unrepaired SEs in abl pre-B cells lacking ATM activity and senataxin protein individually or in combination (Fig. 4B). Collectively, our results suggest that senataxin has important activities during NHEJ that are functionally redundant and uniquely shared with pathways downstream of DNA-PKcs. The lack of synergistic NHEJ defects in ATM-inhibited senataxin-deficient abl pre-B cells also raises the possibility that ATM and senataxin may be epistatic during NHEJ-mediated RAG DSB repair.

### Senataxin helicase activity is required for NHEJ

Senataxin has two structured domains: the N-terminal domain and the helicase domain residing in the C terminus (*26*, *33*). The helicase activity of senataxin is critical for its known biological functions including transcriptional termination, gene expression, and R loop resolution (*26*, *27*, *33*–*35*). We therefore determined the impact of the senataxin helicase domain on RAG DSB repair. To circumvent the lack of reliable antibodies to murine senataxin, we inserted a triple HA epitope tag (3HA) at the 3′ end of both *Setx* alleles in frame with the *Setx* coding sequence in WT abl pre-B cells, termed *Setx^3HA/3HA^* hereafter (fig. S4, A and B). V(D)J recombination in *Setx^3HA/3HA^* abl pre-B cells was comparable to that in untagged WT abl pre-B cells and inactivation of senataxin-3HA in *Setx^3HA/3HA^* abl pre-B cells by CRISPR-Cas9 led to V(D)J recombination defects similar to clonal *Setx*^−/−^ abl pre-B cells upon DNA-PKcs kinase inhibition (fig. S4, A and C, and Fig. 1D).

**Fig. 5.**
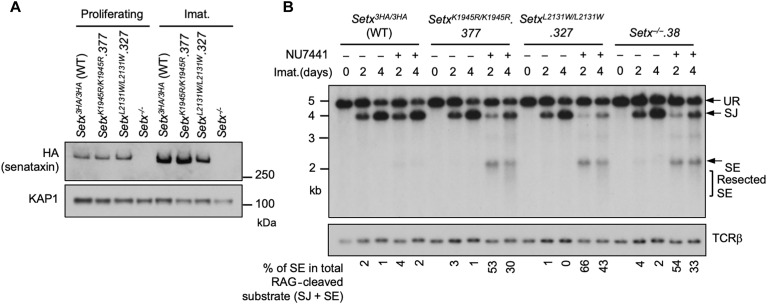
Senataxin helicase activity is required for RAG DSB repair. (**A**) Western blot analysis of cell lysates from proliferating or imatinib-treated *Setx^−/−^*, *Setx^3HA/3HA^*, and *Setx^K1945R/K1945R^* and *Setx^L2131W/L2131W^* abl pre-B cells using HA and KAP1 antibodies. (**B**) Southern blot analysis of Eco RV–digested genomic DNA from cells described in (A) with pMX-DEL^SJ^ and treated with imatinib in the presence or absence of the DNA-PKcs kinase inhibitor NU7441 for the indicated times.

To assess the contribution of the senataxin helicase domain to NHEJ, we introduced homozygous K1945R and L2131W mutations in senataxin that abolish the adenosine triphosphatase (ATPase) and RNA binding activities of senataxin, respectively (*26*, *27*, *33*, *34*). The K1945R and L2132W mutations did not result in a change in the abundance of the mutant proteins when compared to WT senataxin (Fig. 5A and fig. S5, A and C). Upon imatinib treatment, both *Setx^K1945R/K1945R^* and *Setx^L2132W/L2132W^* abl pre-B cells formed SJs to an extent similar to WT and *Setx*^−/−^ abl pre-B cells (Fig. 5B and fig. S5, B and D). However, when these mutant cells were treated with imatinib and the DNA-PKcs kinase inhibitor NU7441, they exhibited a substantial reduction in SJ formation and an increased accumulation of unrepaired SEs (Fig. 5B and fig. S5, B and D), similar to that observed in *Setx*^−/−^ abl pre-B cells (Fig. 2D and fig. S2B). We conclude that an intact helicase function of senataxin is required for NHEJ in the absence of DNA-PKcs kinase activity.

### Senataxin NHEJ function is redundant with the RECQL5 and HLTF helicases

**Fig. 6.**
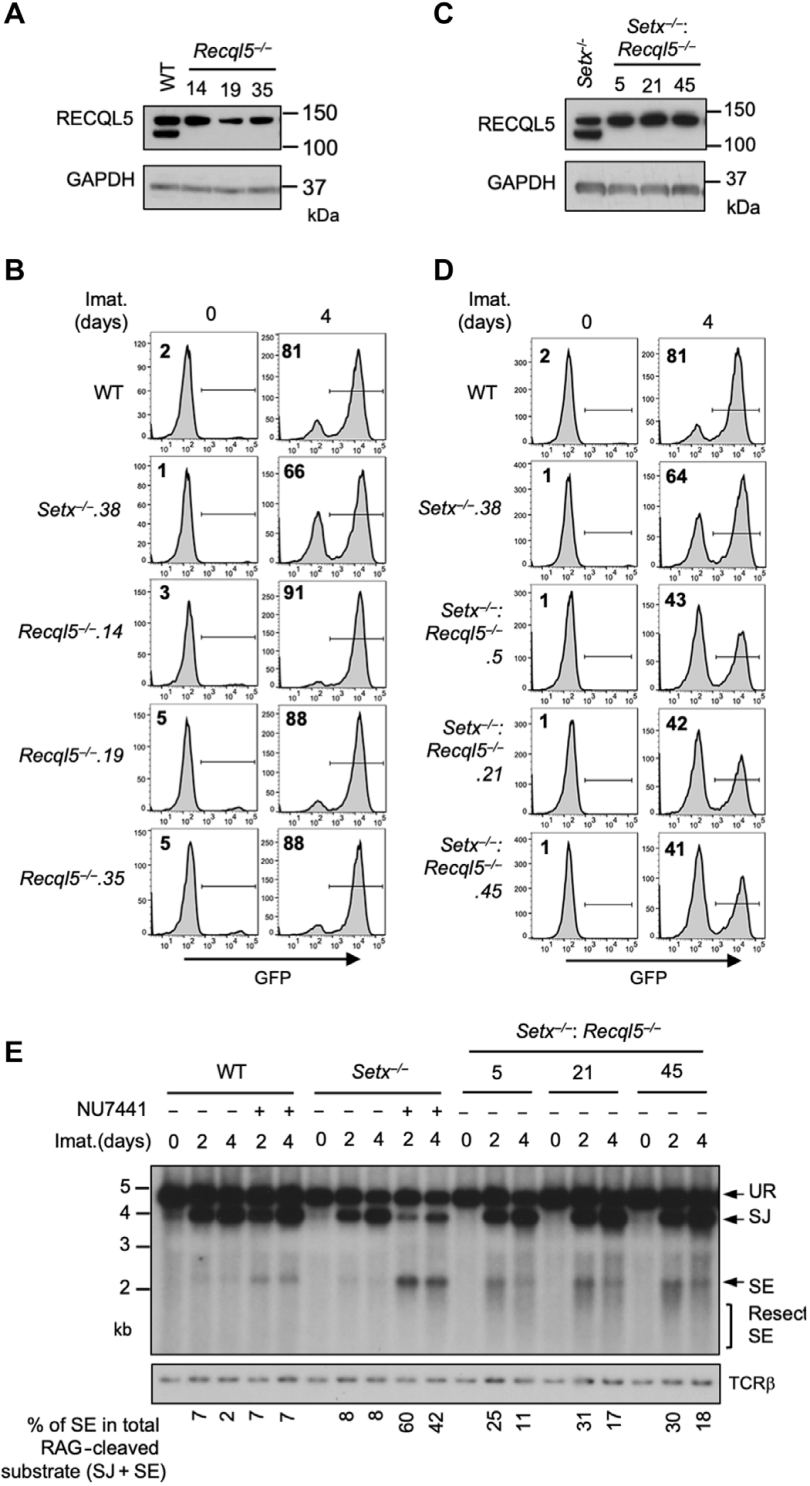
Loss of RECQL5 modestly impairs RAG-DSB repair in *Setx*^−/−^ abl pre-B cells. (**A**) Western blot analysis of cell lysates from WT and three independently isolated *Recql5*^−/−^ abl pre-B cells using RECQL5 and glyceraldehyde-3-phosphate dehydrogenase (GAPDH) antibodies. (**B**) Flow cytometric analysis for GFP expression in cells described in (A) with pMG-INV and treated with imatinib for the indicated times. The percentages of GFP^+^ cells are indicated in the top left corners of the histograms. (**C**) Western blot analysis of cell lysates from *Setx*^−/−^ and three independently isolated *Setx*^−/−^: *Recql5*^−/−^ abl pre-B cells using RECQL5 and GAPDH antibodies. (**D**) Flow cytometric analysis for GFP expression in cells described in (C) treated with imatinib for the indicated times. (**E**) Southern blot analysis of Eco RV–digested genomic DNA from cells described in (D) with pMX-DEL^SJ^ and treated with imatinib in the presence or absence of the DNA-PKcs kinase inhibitor NU7441 for the indicated times.

Our results suggest that senataxin acts in the same pathway as ATM to promote NHEJ when DNA-PKcs is absent. To test whether other helicases might function in NHEJ and be similarly governed by DNA-PKcs, we conducted a genome-wide CRISPR-Cas9 gRNA screen in *Setx*^*−*/−^ abl pre-B cells containing the pMG-INV recombination substrate. This revealed that gRNAs to *Recql5*, encoding the RECQ helicase family member RECQL5, were enriched in the GFP^−^ population of *Setx*^*−*/−^, but not WT, abl pre-B cells (tables S1, S4, and S5). Loss of RECQL5 alone did not have a discernible impact on V(D)J recombination as imatinib-treated *Recql5*^−/−^, and WT cells had similar percentages of GFP^+^ cells (Fig. 6, A and B, and table S3C). Analysis of *Setx*^−/−^: *Recql5*^−/−^ abl pre-B cell lines, however, revealed a moderate but consistent reduction in the percentage of GFP^+^ cells when compared to *Setx*^−/−^ abl pre-B cells (Fig. 6, C and D, and table S3D). This V(D)J recombination deficiency in *Setx*^−/−^: *Recql5*^−/−^ abl pre-B cells is a result of a defect in NHEJ-mediated RAG DSB repair as evidenced by Southern blot analysis of pMX-DEL^SJ^ in these cells revealing accumulation of unrepaired SEs (Fig. 6E). Together, these data show that loss of RECQL5 in *Setx*^−/−^ abl pre-B cells compromises NHEJ.

**Fig. 7.**
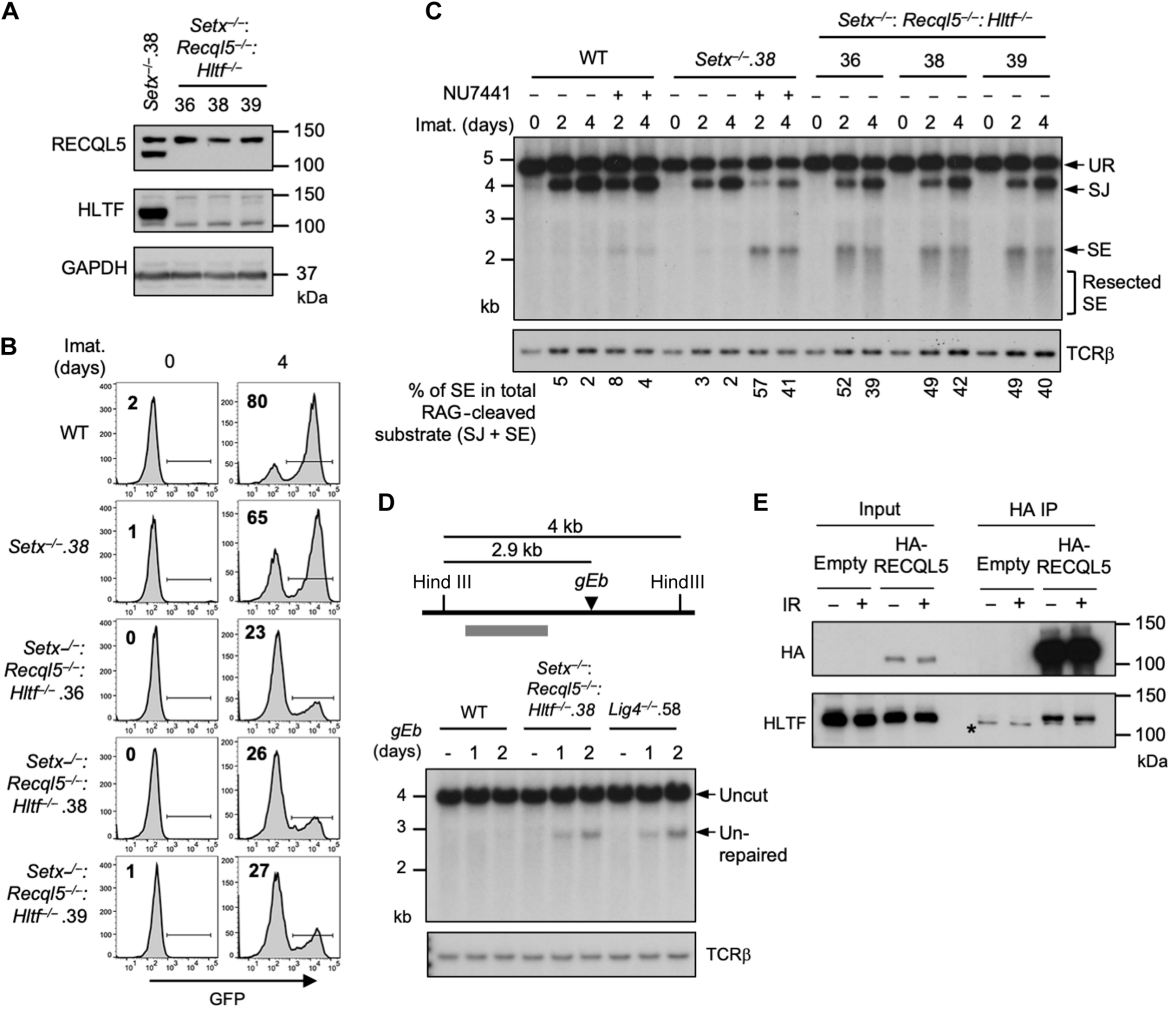
Combined loss of senataxin, RECQL5, and HLTF results in defected RAG DSB repair. (**A**) Western blot analysis of cell lysates from *Setx*^−/−^ and three independently isolated *Setx*^−/−^: *Recql5*^−/−^*: Hltf*^−/−^ abl pre-B cells using RECQL5, HLT, and GAPDH antibodies. (**B**) Flow cytometric analysis for GFP expression in WT, *Setx*^−/−^ and *Setx*^−/−^: *Recql5*^−/−^*: Hltf*^−/−^ abl pre-B cells with pMG-INV and treated with imatinib for the indicated times. The percentages of GFP^+^ cells are indicated in the top left corners of the histograms. (**C**) Southern blot analysis of *EcoR*V-digested genomic DNA from abl pre-B cells described in (B) with pMX-DE^SJ^ and treated with imatinib in the presence or absence of the DNA-PKcs kinase inhibitor NU7441 for the indicated times. (**D**) Schematic for Southern blot analysis of Cas9 DSB repair in the Eb region of the *Tcrb* gene. The *gEb* binding and restriction enzyme Hind III cutting locations are shown. The Eb probe is indicated as the gray bar (top). Southern blot analysis of Hind III–digested genomic DNA from imatinib-treated WT, *Setx*^−/−^: *Recql5*^−/−^*: Hltf*^−/−^, and *Lig4*^−/−^ abl pre-B cells expressing Cas9 and *gEb* using the Eb probe (bottom). (**E**) Western blot analysis of HLTF co-immunoprecipitation with ectopically expressed HA-RECQL5 in imatinib-treated abl pre-B cell lysate with HA and HLTF antibodies. The asterisk indicates the nonspecific recognizing bands by the HLTF antibody. IP, immunoprecipitation.

We reasoned that the modest impact of combined loss of senataxin and RECQL5 on NHEJ-mediated RAG repair, as compared to co-inactivation of senataxin and DNA-PKcs, could be due to the activities of additional helicases. A genome-wide CRISPR-Cas9 gRNA screen in imatinib-treated *Setx*^−/−^*: Recql5*^−/−^ abl pre-B cells identified multiple gRNAs to *Hltf*, encoding the double-strand DNA translocase and chromatin remodeler HLTF, being enriched in the GFP^−^ population (table S6). Loss of HLTF, in WT or *Setx*^−/−^ abl pre-B cells, had no effect on V(D)J recombination as revealed by flow cytometric analysis of cells containing pMG-INV (fig. S6 and table S3, E and F). Moreover, abl pre-B cells lacking both RECQL5 and HLFT also exhibited normal V(D)J recombination (fig. S7 and table S3G). However, *Setx*^−/−^*: Recql5*^−/−^: *Hltf*^−/−^ abl pre-B cells with integrated pMG-INV recombination substrate exhibited a substantial reduction in GFP^+^ cells upon induction of V(D)J recombination when compared to *Setx*^−/−^ and *Setx*^−/−^*: Recql5*^−/−^ abl pre-B cells (Figs. 7, A and B, and 6D, and table S3H). Reduced SJ formation and increasing levels of unrepaired SEs were also observed upon Southern blot analysis of pMX-DEL^SJ^ indicating a strong NHEJ defect in *Setx*^−/−^*: Recql5*^−/−^: *Hltf*^−/−^ abl pre-B cells (Fig. 7C). To determine whether the NHEJ defect observed in *Setx*^−/−^*: Recql5*^−/−^: *Hltf*^−/−^ abl pre-B cells is unique to the repair of RAG DSB or also applicable to other types of DSBs, we monitored the repair of Cas9-induced DSBs at the enhancer Eb region of the *Tcrb* locus (*36*). Unrepaired Cas9 DSBs were observed in *Lig4*^−/−^ and *Setx*^−/−^*: Recql5*^−/−^: *Hltf*^−/−^ but not in WT abl pre-B cells (Fig. 7D and fig. S8, A to C). However, consistent with recent studies reporting the requirement of HLTF in the repair of Cas9-induced DSBs, we also detected unrepaired Cas9 DSBs in *Hltf*^−/−^ abl pre-B cells (fig. S8, D to F) (*37*, *38*). Therefore, the sensitivity our current assay prevents us to conclude whether combined loss of all three proteins leads to a stronger defect in the repair of Cas9 DSBs than loss of HLTF alone. We additionally determined the sensitivity to radiation of G_0_/G_1_-arrested ab pre-B cells. As expected, *Lig4*^−/−^ abl pre-B cells were significantly more sensitive than WT abl pre-B cells to a range of radiation. However, no difference in radiosensitivity was observed in DNA-PKcs inhibitor-treated WT abl pre-B cells, *Setx*^−/−^, and *Setx^−/−^*: *Recql5*^−/−^: *Hltf*^*−*/−^ abl pre-B cells when compared to WT abl pre-B cells. DNA-PKcs inhibitor–treated *Setx*^−/−^ abl pre-B cells were slightly more sensitive to radiation treatment than WT abl pre-B cells, although the difference fell short to be statistically significant (fig. S9). Collectively, these data indicate that senataxin, RECQL5, and/or HLTF have redundant functions in the repair of some DSBs, such as RAG- and Cas9-induced DSBs, but may be dispensable for the resolution of others, such as those induced by radiation. We noted that the NHEJ defect during V(D)J recombination in the *Setx*^−/−^: *Recql5*^−/−^: *Hltf*^−/−^ abl pre-B cells is unlikely caused by deregulated cell proliferation or reduced viability upon imatinib treatment. The 5-bromo-2′-deoxyuridine (BrdU) labeling of the proliferating triple deficient (and all other single- and double-deficient) abl pre-B cells revealed comparable cell cycle distribution to WT abl pre-B cells (fig. S10). All the mutant abl pre-B cells arrested in G_1_-G_0_ as efficiently as WT cells upon imatinib treatment (fig. S11). Moreover, the viability of the single or combined helicase-deficient abl pre-B cells in proliferation or after imatinib treatment was not different from that of WT abl pre-B cells (figs. S12 and S13). We conclude that the RECQL5 and HLTF helicases function redundantly with senataxin during the NHEJ-mediated RAG DSB repair. In this regard, to test whether RECQL5 and HLTF could exert their function as a complex, we conducted immunoprecipitation assay in imatinib-treated abl pre-B cells expressing hemagglutinin (HA)–tagged RECQL5. Our result showed that in the HA antibody immunocomplex, both HA-RECQL5 and endogenous HLTF could be detected in a DNA damage-independent manner, indicating that a fraction of RECQL5 and HLTF constitutively associate with each other in cells (Fig. 7E).

### ATM activity is required for RAG DSB repair in *Recql5*^−/−^: *Hltf*^*−*/−^ abl pre-B cells

**Fig. 8.**
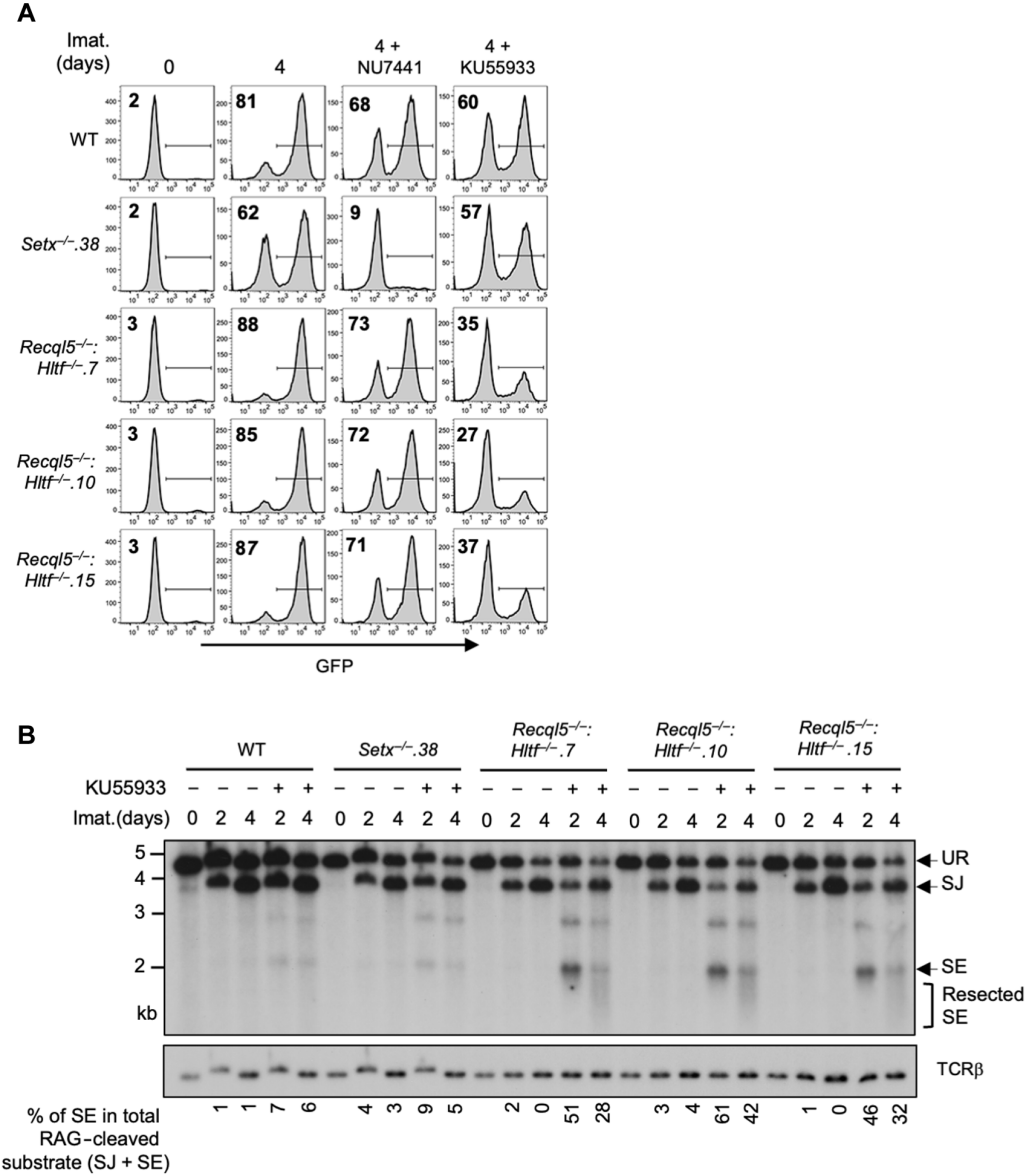
ATM activity is required for RAG DSB repair in *Recql5*^−/−^: *Hltf*^*−*/−^ abl pre-B cells. (**A**) Flow cytometric analysis for GFP expression from pMG-INV in WT, *Setx*^*−*/−^, and three independently isolated *Recql5*^−/−^: *Hltf*^*−*/−^ abl pre-B cell lines treated with imatinib in the presence or absence of the DNA-PKcs kinase inhibitor NU7441 or the ATM kinase inhibitor KU55933 for the indicated times. The percentages of GFP^+^ cells are indicated in the top left corners of the histograms. (**B**) Southern blot analysis of *EcoR*V-digested genomic DNA from cells described in (A) with pMX-DE^SJ^ and treated with imatinib in the presence or absence of KU55933 for the indicated times.

Our results thus far suggest that two pathways, one regulated by senataxin and the other by RECQL5 and HLTF, redundantly support NHEJ-mediated RAG DSB repair. Moreover, that inhibition of the kinase activity of DNA-PKcs, but not ATM, uniquely impairs RAG DSB repair in *Setx*^−/−^ abl pre-B cells suggests an epistatic relationship between ATM and senataxin (Figs. 2 and 4, and fig. S2). We next determined whether DNA-PKcs could function with RECQL5 and HLTF in a similar manner. Inhibition of DNA-PKcs kinase activity by NU7441, despite strongly blocking V(D)J recombination in *Setx*^−/−^ abl pre-B cells, only had modest effects in *Recql5^−/−^*, *Hltf^−/−^*, and *Recql5*^−/−^: *Hltf*^−/−^ abl pre-B cells as measured by GFP expression from pMG-INV in a flow cytometric assay (Fig. 8A; figs. S6B and S14; and tables S3, A, C, E, and I). On the contrary, inhibition of ATM kinase activity with KU55933 moderately decreased V(D)J recombination in WT, *Setx*^−/−^, *Recql5* ^−/−^, and *Hltf^−/−^*abl pre-B cells but substantially reduced the percentage of GFP^+^ cells in *Recql5*^−/−^: *Hltf*^−/−^ abl pre-B cells (Figs. 4A and 8A; figs. S6B and S14; and tables S3, B, C, E, and G). Southern blot analysis of the recombined pMX-DEL^SJ^ substrate in KU55933-treated *Recql5*^−/−^: *Hltf*^−/−^ abl pre-B cells also revealed the accumulation of unrepaired SE fragments, indicating defective RAG DSB repair in these cells (Fig. 8B). Together, these results suggest that ATM may regulate senataxin activity and DNA-PKcs may regulate the activities of RECQL5 and HLTF to promote NHEJ-mediated DSB repair.

We noted that the V(D)J recombination deficiency, as determined by flow cytometric analysis of GFP expression from the recombined pMG-INV substrate, in *Setx*^−/−^: *Recql5*^−/−^: *Hltf*^−/−^ and ATM-inhibited *Recql5*^−/−^: *Hltf*^−/−^ abl pre-B cells was less severe than that in DNA-PKcs–inhibited abl pre-B cells also lacking ATM activity or senataxin (Figs. 1D, 7B, and 8A, and fig. S15). In addition, treating *Setx*^−/−^: *Recql5*^−/−^: *Hltf*^−/−^ abl pre-B cells with the DNA-PKcs inhibitor NU7441 further reduced the recombination of the pMG-INV substrate in the flow cytometric analysis (fig. S16A and table S3J). DNA-PKcs inhibition in *Setx*^−/−^: *Recql5*^−/−^: *Hltf*^−/−^ abl pre-B cells also led to additional loss in SJ formation, accompanied by increased accumulation of unrepaired SEs in Southern blot analysis (fig. S16B). Thus, in addition to RECQL5 and HLTF, additional factors likely also function in the DNA-PKcs–dependent pathway to promote NHEJ.

### Partial inhibition of RNA polymerase II activity improves RAG DSB repair in helicase-deficient abl pre-B cells

**Fig. 9.**
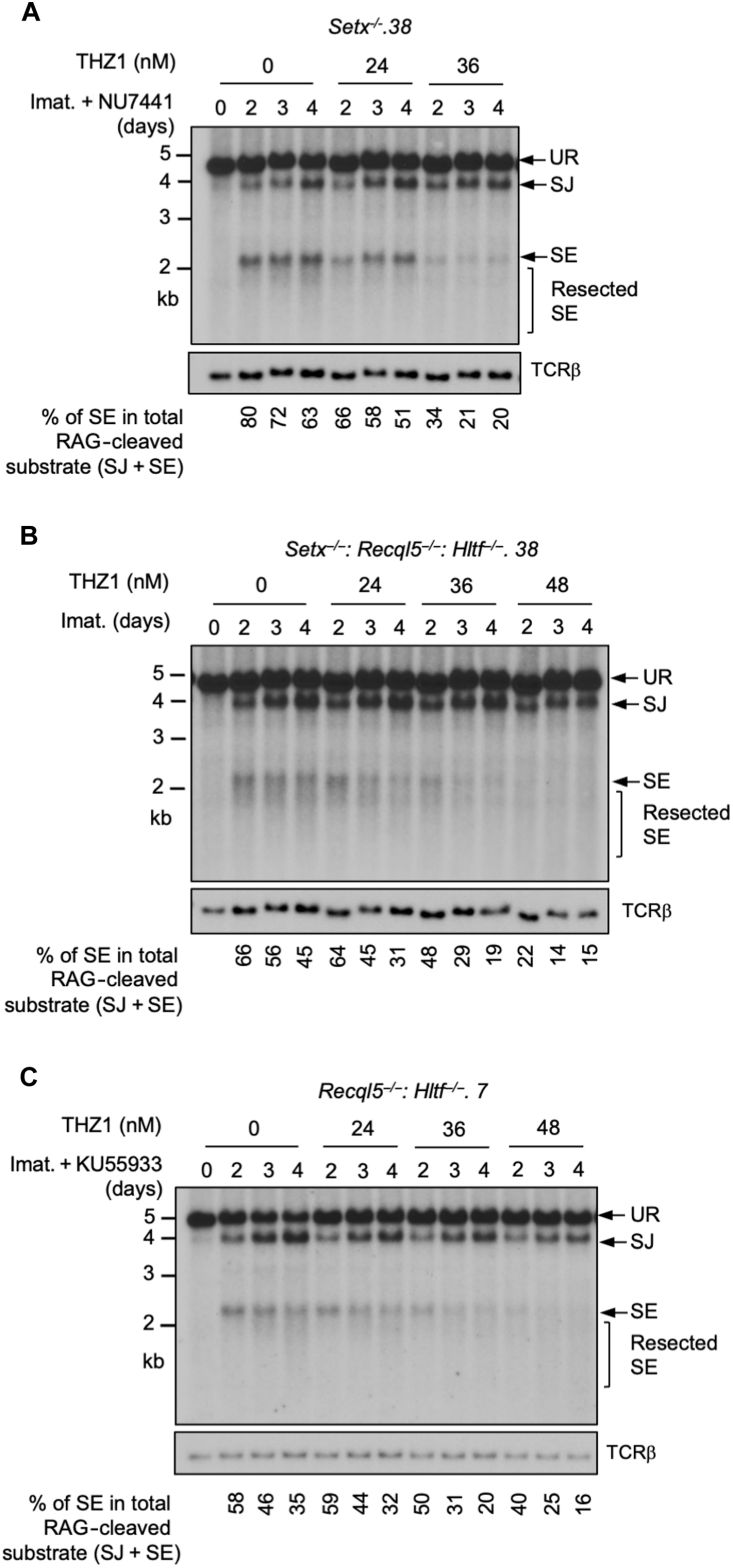
Partial inhibition of RNA polymerase II improves NHEJ-mediated RAG DSB repair in helicase-deficient abl pre-B cells. Southern blot analysis of Eco RV–digested genomic DNA from pMX-DEL^SJ^ containing, imatinib treated *Setx*^−/−^ (with the DNA-PKcs kinase inhibitor NU7441) (**A**), *Setx*^−/−^: *Recql5*^−/−^*: Hltf*^−/−^ (**B**), and *Recql5*^−/−^*: Hltf*^−/−^ (with the ATM kinase inhibitor KU55933) (**C**) abl pre-B cells with increasing concentrations (24, 36, and 48 nM) of the CDK7 inhibitor THZ1 for the indicated times.

During V(D)J recombination, RAG DSBs are generated and repaired in actively transcribed chromatin regions. Given mounting evidence demonstrating the impact of transcription on DSB repair, we tested whether the NHEJ defect seen in our mutant abl pre-B cells is transcription dependent (*39*, *40*). To this end, *Setx*^*−*/−^ abl pre-B cells were exposed to low doses of THZ1, a highly selective inhibitor to the RNA pol II carboxy-terminal domain (CTD) kinase CDK7, 24 hours after imatinib and DNA-PKcs inhibitor treatment (*41*). In Southern blot analysis, we observed that with increasing concentrations of THZ1, the intensity of the restriction fragments corresponding to SEs decreased. Quantifications showed that the percentage of unrepaired SEs in the total amount of the recombination substrate cleaved by RAG (the sum of intensities of restriction fragments to repaired SJs and unrepaired SEs) was reduced in cells treated with increasing concentrations of THZ1 (Fig. 9A and fig. S17A). Similar observations were made in *Setx*^−/−^*: Recql5*^−/−^: *Hltf*^−/−^ and ATM kinase inhibitor–treated *Recql5*^−/−^: *Hltf*^−/−^ abl pre-B cells (Fig. 9, B and C, and fig. S17, B and C). We noted that the sameTHZ1 treatments in abl pre-B cells with normal NHEJ-mediated RAG DSB repair did not have demonstrable effects on SJ formation with the exception of DNA-PKcs-inhibited *Recql5*^−/−^: *Hltf*^−/−^ abl pre-B cells, where RAG-mediated cleavage of the pMX-DEL^SJ^ substrate was strongly inhibited even at the lowest concentration of THZ1 (fig. S18). These results indicate that partial inhibition of RNA pol II activity in abl pre-B cells can partly rescue NHEJ defects due to combined deficiency of senataxin and DNA-PKcs; co-inactivation of ATM, RECQL5, and HLTF; and loss of senataxin, RECQL5, and HLTF.

### Aberrant DNA end joining in abl pre-B cells deficient in senataxin, RECQL5, and HLTF

**Fig. 10.**
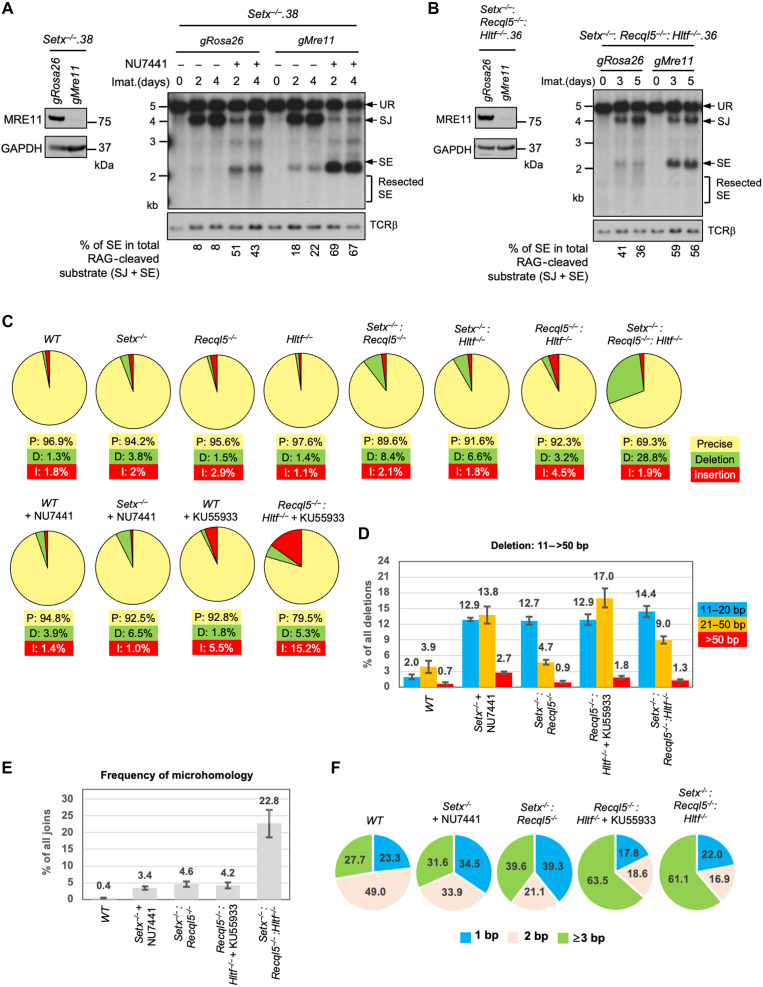
Aberrant DNA end joining in abl pre-B cells deficient in senataxin, RECQL5, and HLTF. (**A** and **B**) Left: Western blot analysis of cell lysate from *Setx*^−/−^ (A) or *Setx*^−/−^: *Recql5*^−/−^*: Hltf*^−/−^ (B) abl pre-B cells expressing Cas9 and gRNAs to *Rosa26* (*gRosa26*) or *Mre11* (*gMre11*) using MRE11 and GAPDH antibodies. Right: Southern blot analysis of Eco RV–digested genomic DNA from the aforementioned cells with pMX-DEL^SJ^ and treated with imatinib in the presence or absence of the DNA-PKcs kinase inhibitor NU7441 for the indicated times. (**C**) Percentages of precise joints (P, yellow), joints with deletions (D, green), and joints with insertions (I, red) in SJs amplified from pMG-INV in the indicated abl pre-B cells after imatinib treatment for 4 days in the presence or absence of DNA-PKcs kinase inhibitor NU7441 or ATM kinase inhibitor KU55933. (**D**) Percentages of long (11 to >50 bp) nucleotide loss in deletion-containing SJs amplified from pMG-INV in the indicated abl pre-B cell lines. The numbers above each bar indicate the average percentages of SJ sequences with the specific length distributions of deletion. (**E**) Percentages of SJs with microhomology at joining junctions in the indicated abl pre-B cell lines with the average percentages of SJs with microhomology shown on top of each bar. (**F**) Microhomology length distribution (% of all microhomology) of the SJs shown in (F). Number of samples sequenced per genotype: WT: 10, WT + NU7441: 3, WT + KU55933: 3, *Setx^−/−^*: 6, *Setx*^−/−^ + NU7441: 3, *Recql5^−/−^*: 3, *Hltf*^−/−^: 3, *Setx*^−/−^: *Recql5*^−/−^: 4, *Setx*^−/−^: *Hltf*^−/−^: 3, *Recql5*^−/−^: *Hltf^−/−^*: 3, *Recql5*^−/−^: *Hltf*^*−*/−^ + KU55933: 3, and *Setx*^−/−^: *Recql5*^−/−^: *Hltf*^−/−^: 4.

In *Setx*-deficient abl pre-B cells treated with DNA-PKcs inhibitor, not only did we observe the accumulation of unrepaired SEs and CEs in Southern blot analyses, but we also found that the restriction fragments of these unrepaired DNA ends were heterogeneous in size, suggesting that they were aberrantly resected (Figs. 2 and 5 and figs. S2 and S5) (*36*, *42*, *43*). Similar observations were made in *Setx*^−/−^*: Recql5*^−/−^, *Setx*^−/−^*: Recql5*^−/−^: *Hltf*^−/−^, and ATM kinase inhibitor–treated *Recql5*^−/−^: *Hltf*^−/−^ abl pre-B cells (Figs. 6E, 7C, and 8B). When MRE11, which initiates DNA resection, was depleted by CRISPR-Cas9, the heterogeneity in size of SE bands was markedly reduced and a single strong band appeared, consistent with the notion that the unrepaired DNA ends are resected in these cells (Fig. 10, A and B) (*44*). We note that higher levels of unrepaired SEs were observed in *Setx*^−/−^ abl pre-B cells (without NU7441 treatment) expressing *gMre11* compared to those expressing the control *gRosa26*. (Fig. 10A). As MRE11 deficiency alone does not have demonstrable effect on the repair of SEs in WT abl pre-B cells, this observation may indicate a potential functional redundancy between MRE11 and senataxin during NHEJ (*45*). The MRE11-depleted cells are expected to be defective in ATM activation. Therefore, the strong loss of SJ formation in NU7441-treated *Setx*^−/−^ abl pre-B cells expressing *gMre11* is likely due to the combined inactivation of ATM and DNA-PKcs in these cells (Fig. 10A).

During NHEJ-mediated DSB repair, the resection of DNA ends and the resulting short single-stranded overhangs could lead to aberrant homology-mediated joining, which frequently results in nucleotide loss. During V(D)J recombination, accurate SJ formation occurs with only a small fraction of SJs losing one or two nucleotides (*46*). To survey the structural variants of SJs in cells deficient in ATM, DNA-PKcs, senataxin, RECQL5, and HLTF, we PCR-amplified and sequenced pMG-INV SJs using genomic DNA extracted from different abl pre-B cell lines. As expected, most of the SJs in WT abl pre-B cells were formed precisely (~97%) with a small percentage of nucleotide loss (deletion) and gain (insertion). In addition, individual inhibition of ATM or DNA-PKcs kinase activities or singular loss of senataxin, RECQL5, or HLTF also did not have strong impact on the fidelity of repaired SJs (Fig. 10C and table S7). However, the inhibition of DNA-PKcs in *Setx^−/−^* abl pre-B cells resulted in an increase in the frequency of SJs containing deletions (Fig. 10C and table S7). Higher levels of SJs with deletions were also observed in *Setx^−/−^: Recql5*^−/−^, ATM inhibitor-treated *Recql5*^−/−^: *Hltf*^*−*/−^, and *Setx*^−/−^*: Recql5*^−/−^: *Hltf*^−/−^ abl pre-B cells, where RAG DSB repair defects were observed (Fig. 10C and table S7). Most of the deletions are shorter than 10 base pairs (bp); however, longer deletions (>10 bp) are more frequent in the mutants in which higher fractions of SJs contain deletions (Fig. 10D and table S7). Notably, in addition to deletions, a substantial fraction of SJs in ATM-inhibited *Recql5*^−/−^: *Hltf*^*−*/−^ abl pre-B cells also contained insertions (Fig. 10C and table S7).

Sequence analyses revealed that less than 1% of SJs in WT abl pre-B cells contained microhomology at SJ junctions, whereas 23% of SJs from *Setx*^−/−^*: Recql5*^−/−^: *Hltf*^−/−^ abl pre-B cells exhibited microhomology with 60% of these exhibiting 3 bp or more of microhomology (Fig. 10, E and F). We conclude that combined deficiency of DNA-PKcs and senataxin; co-inactivation of ATM, RECQL5, and HLTF; and loss of senataxin, RCQL5, and HLTF result in an increase in aberrant joining during NHEJ and that this joining is more dependent on the use of microhomologies.

## DISCUSSION

In this study, we exploited V(D)J recombination, a process requiring NHEJ for proper completion, to investigate novel factors that function in ATM- or DNA-PKcs–dependent pathways to promote NHEJ. Given the prominent roles of DNA-PKcs in NHEJ, we initially conducted a CRISPR screen for genes required for efficient V(D)J recombination in cells treated with the DNA-PKcs inhibitor NU7441. Genetic ablation of DNA-PKcs kinase activity or autophosphorylation has been shown to impede NHEJ, including the repair of RAG DSBs, and prolong the association of mutant DNA-PKcs proteins with broken DSB ends. These results indicate that DNA-PKcs kinase–mediated autophosphorylation may be required for the release of the protein from DSBs or conformational changes of the active kinase to render DNA ends accessible to the ligation complex (*30*–*32*). In contrast to kinase-inactivating mutations in DNA-PKcs, the DNA-PKcs inhibition by chemical inhibitors does not have conspicuous effect in the repair of RAG DSBs during V(D)J recombination (*20*, *21*, *30*). The nature of the difference between genetic and chemical inactivation of DNA-PKcs on NHEJ is currently unclear. We speculate that it is possible that the inhibition of DNA-PKcs by inhibitors is incomplete, and the remaining activity is sufficient for end joining in this context (*47*). In this regard, the DNA-PKcs inhibitor NU7441, and likely other inhibitors, binds and inhibits DNA-PKcs reversibly (*48*). The exchange between NU7441-bound and NU7441-unbound states may also allow NHEJ to occur at a slower kinetics.

Our findings collectively depict two functionally redundant genetic pathways supporting NHEJ-mediated RAG DSB repair during V(D)J recombination with one mediated by senataxin and ATM and the other by RECQL5, HLTF, and DNA-PKcs. Senataxin and RECQL5 have been implicated in DSB repair, while the known functions of HLTF are largely restricted to the response to replicative stress (*49*–*56*). Senataxin, RECQL5, and HLTF are all able to resolve unique DNA structures in vivo or in vitro. Senataxin can unwind RNA:DNA hybrids, including R loops, and loss of senataxin can lead to R loop accumulation at DSBs (*50*). The helicase activity of RECQL5 is important for resolving Holiday junction-like structures in vitro, and both RECQL5 and HLTF have recently been shown to promote the resolution of noncanonical nucleic acid structures G-quadruplexes (G4s) (*57*–*59*). Given the activities of these helicases in untangling unique nucleic acid structures, it is possible that they may function redundantly to ensure that inhibitory structures do not accumulate at DSB ends to impede the recruitment of NHEJ factors or prevent DNA end ligation (*52*). Notably, RECQL5 and HLTF can also remodel or remove chromatin-bound proteins to facilitate proper duplication of common fragile sites in mitosis and replication fork reversal, respectively (*60*, *61*). Moreover, recent studies demonstrated that HLTF disrupts the Cas9-DNA postcleavage complex before DSB repair (*37*, *38*). We observed that in G_0_-arrested *Hltf*^−/−^ and *Setx*^−/−^*: Recql5*^−/−^: *Hltf*^−/−^ abl pre-B cells, unrepaired Cas9-induced DSBs persisted as in *Lig4*^−/−^ abl pre-B cells in Southern blot analysis (Fig. 7D and figs. S8F). Additional assays will be needed to quantitatively investigate whether senataxin, RECQL5, and HLTF all contribute to the repair of Cas9 DSBs. Therefore, in addition to inhibitory DNA structures, it is possible that at least some of these proteins could function for timely removal of proteins bound to DSB ends. Along these lines, RNA pol II–dependent transcription is often accompanied by noncanonical nucleic acid structures such as R loops and G4s, and aberrant accumulation of these structures leads to DNA damage (*62*, *63*). Our finding that RAG DSB repair is more efficient when RNA pol II activity is partially suppressed raises the possibility of a transcription-related conflict with NHEJ that is normally prevented by concerted activities of senataxin, RECQL5, and HLTF. The identity of the molecular blockades that require these helicases and translocase for resolution during DSB repair remains to be determined. We note that V(D)J recombination occurs in transcriptionally active germline alleles, raising the possibility that mechanisms entailing factors such as the helicases and translocase described here may exist to ensure that the proper completion of joining process is not affected by potential transcription-related obstacles. Along the line, our observation that DNA-PKcs–inhibited *Setx^−/−^* and *Setx*^−/−^*: Recql5*^−/−^: *Hltf*^−/−^ abl pre-B cells demonstrated comparable radiosensitivity to WT, as opposed to *Lig4*^−/−^, abl pre-B cells indicates that the senataxin- and RECQL5/HLTF-mediated pathways likely function to resolve a subset of DSBs with unique features, rather than being broadly required for NHEJ of all DSBs.

ATM and DNA-PKcs are known to phosphorylate many unique and overlapping protein targets with diverse functions that promote the DNA damage response (*18*, *64*). This could, in part, explain the observation that ATM and DNA-PKcs promote NHEJ through pathways with overlapping functions such that cells retain considerable DNA DSB repair capability when one of these kinases is inhibited (*19*–*21*). Our results indicate that ATM and DNA-PKcs support RAG DSB repair through proteins with distinct helicase or translocase activities in a manner in which ATM appears epistatic to senataxin and DNA-PKcs appears epistatic to RECQL5 and HLTF. This is supported by co-inactivation of ATM, RECQL5, and HLTF, or combined deficiency in DNA-PKcs and senataxin that leads to defective RAG DSB repair. In addition, inactivation of DNA-PKcs and ATM in *Recql5*^−/−^: *Hltf*^−/−^ and *Setx*^−/−^ abl pre-B cells, respectively, did not result in additional RAG DSB repair defects (Figs. 1, 2, 4, and 8, and fig. S2). We noted that DNA damage repair defects in the setting of combined deficiency in DNA-PKcs and senataxin have also been observed in fibroblasts derived from patients with spinal muscular atrophy (SMA) and neurons from SMA mice, where the expression of senataxin and DNA-PKcs is compromised and elevated levels of endogenous DNA damage marked by γH2AX persist (*65*), raising the possibility that the kinase-helicase regulatory pathways described here likely function in genome maintenance in a broader context. The molecular mechanisms by which ATM and DNA-PKcs regulate senataxin, RECLQ5, and HLTF during RAG DSB repair remain to be determined. It is to be noted that DNA-PKcs was reported to associate with senataxin in a previous study that also showed that senataxin localization to DSBs was diminished upon inhibition of the kinase activities of ATM and DNA-PKcs in proliferating HeLa cells (*49*). In addition, senataxin was recently found to undergo ataxia telangiectasia and Rad3–dependent phosphorylation, and this phosphorylation was reported to be important for senataxin association with sex chromosomes during meiosis (*66*). Therefore, senataxin can be regulated by multiple DDR kinases to execute various biological functions in a cell type–dependent manner to maintain genome integrity. It remains to be investigated whether senataxin is directly modified by these kinases and if phosphorylation fine-tunes senataxin activity. Little is known regarding whether or how DDR kinases regulate the function of RECQL5 and HLTF in response to DNA damage or replicative stress. Our results provide supporting evidence for this possibility that warrants further investigation.

One of the major factors that dictate the choice of DSB repair pathways between NHEJ and HR is the structure of the broken DNA ends. Extensive resection of DNA ends generates long single-strand overhangs that promote HR and limits NHEJ (*67*). To ensure that DNA DSBs to be repaired by NHEJ are not aberrantly processed, multiple DNA end protection mechanisms exist to counter the nucleolytic activity (*68*, *69*). We observed that the unrepaired SEs in *Setx*^−/−^*: Recql5*^−/−^: *Hltf*^−/−^ abl pre-B cells were resected (Fig. 7C). Similar observations were also made in *Setx*^−/−^*: Recql5*^−/−^ abl pre-B cells (Fig. 6E), suggesting that at least senataxin and/or RECQL5 may suppress aberrant resection of DSBs before their joining by NHEJ. Senataxin deficiency in yeast and human cells has been reported to promote resection at DSBs (*51*, *70*). In addition, senataxin was found to interact with MRE11 in human cells, although it is not clear whether this interaction influences MRE11 nuclease activity at DSBs (*49*). In a large-scale proteomics study, senataxin was identified as a potential interactor of the pro-resection Breast Cancer 1 (BRCA1) and the DNA end protector 53BP1. The functional significance of these interactions remains to be determined (*71*). MRE11 can also associate with RECQL5 in human cells and mediates the recruitment of RECQL5 to DSBs. Moreover, it was shown in an in vitro assay, RECQL5 could inhibit the 3′ to 5′ exonuclease activity of MRE11 (*54*). Whether RECQL5 also negatively regulates MRE11 exonuclease activity in cells is yet to be investigated. No existing evidence suggests that HLTF may directly or indirectly influence nucleases or DNA end protection proteins. We found in the survey of SJ structural variants that the loss of HLTF in *Setx*^−/−^*: Recql5*^−/−^ abl pre-B cells significantly elevated the frequency of SJs with deletions, which most likely come from nuclease-processed SEs. The lengths of deletions are also longer in *Setx*^−/−^*: Recql5*^−/−^: *Hltf*^−/−^ compared to *Setx*^−/−^*: Recql5*^−/−^: abl pre-B cells (Fig. 10, C and D). These results suggest that HLTF may also function to limit nucleolytic processing of DSBs, especially in conditions where DNA ends are prone to be resected (i.e. cells lacking senataxin and RECQL5). The presence of increased deletions and microhomology at repaired junctions has also be reported in NHEJ-deficient KU70-deficient abl pre B cells, where alternative end joining (A-EJ) mechanisms promote the repair of RAG DSBs (*72*). Our observation of similar mutagenic features raises the possibility that A-EJ pathways could be responsible for at least a fraction of the end joining activities observed in DNA-PKcs–inhibited *Setx*^−/−^, ATM-inhibited *Recql5^−/−^*: *Hltf*^*−*/−^, and/or *Setx*^−/−^*: Recql5*^−/−^: *Hltf*^−/−^ abl pre-B cells.

Together, our study demonstrates that a group of helicases and a translocase function redundantly to promote proper completion of NHEJ-dependent RAG DSB repair in an ATM- or DNA-PKcs–dependent manner. It is conceivable that this collection of helicases and kinases may also be important for NHEJ in other contexts such as DSB repair in G_1_-G_0_ phases of the cell cycle or the repair of DSBs in transcriptionally active regions including those generated during class switch recombination.

## MATERIALS AND METHODS

### Cell cultures and cell line generation

WT (M63.1: iCas9.302: pMG-INV 36) abl pre-B cells with chromosomally integrated pMG-INV recombination substrate, and pCW-Cas9 (Addgene, #50661) was described previously (*29*). Briefly, the stable integration of pCW-Cas9 was selected by puromycin (2 μg/ml) for 6–7 days. Inducible and homogenous expression of 3XFLAG-Cas9 upon dox treatment in selected clonal cell lines was confirmed by flow cytometry. The retroviral pMG-INV recombination substrate was then transduced to the resulting cell line WT (M63.1: iCas9.302), and stable transduced cells were selected by the expression of Thy1.2 marker and subcloned by serial dilution. Subclones with a single copy of integrated pMG-INV were identified by Southern blot. The subclones with a single copy of integrated pMG-INV were further tested for the ability to undergo efficient V(D)J recombination upon imatinib treatment by flow cytometry analysis of GFP expression. WT (M63.1:iCas9.302: pMG-INV 36) was found to inducibly and homogenously express Cas9, contain a single copy integrated pMG-INV and undergo robust V(D)J recombination (>80% GFP^+^ cells 4 days after imatinib treatment) and was selected for further analysis and the generation of mutant cell lines used in this work. B.WT abl pre-B cells were immortalized from primary pre-B cells harvested from mice obtained from the Jackson Laboratory (strain #029415) following the same procedure (*29*). To generate clonal abl pre-B cell lines deficient in genes investigated in this work, abl pre-B cells inducibly expressing Cas9 were treated with doxycycline (2 μg/ml) for 2 days to induce Cas9 expression, followed by electroporation with the gRNA-expressing lentiviral vector pKLV-gRNA (gene-specific)–hCD2 using the 4D Nucleofector (Lonza) with the SG Cell Line 4D-Nucleofector X Kit L and the pulse code CM147. Twenty-four hours after electroporation, gRNA-expressing cells were sorted based on hCD2 expression by magnetic-activated cell sorting (MACS) cell separators and MS columns (Miltenyi Biotec). Clonal-deficient mutant cells were obtained by limiting dilutions, and the inactivation of targeted genes in the resulting cell lines was verified by Sanger sequencing (*Setx*^−/−^) or Western blot (*Recql5^−/−^*, *Hltf*^−/−^, and *Prkdc*^−/−^). To sequence mutated *Setx* alleles, amplicons containing *Setx* exon 4, where the *gSetx* targets, were generated by polymerase chain reaction (PCR) and cloned into the pCR-Blunt-II-TOPO vector (Invitrogen) for sequencing.

To generate abl pre-B cell lines with a 3HA epitope tag in-frame inserted at the 3′ end of the endogenous *Setx* coding sequence, a recombination donor containing the 3HA coding sequence flanked by ~800-bp genomic sequences immediately 5′ or 3′ to the stop codon of *Setx* was generated by PCR, cloned into pCR-Blunt-II-TOPO vector (Invitrogen), and verified by sequencing. The sequence of a gRNA targeting near the *Setx* stop codon was included at the 5′ and 3′ end of the recombination donor DNA for liberating the linear donor DNA fragment upon transfection into abl pre-B cells expressing the same gRNA and Cas9. The recombination donor and the gRNA expressing plasmids were transfected to WT abl pre-B cells expressing Cas9 using the 4D Nucleofector (Lonza) as described above. The resulting cells, after selected for hCD2 expression from the gRNA expressing vector, were subjected to limiting dilution to isolate single-cell clones. Clonal *Setx^3HA/3HA^* abl pre-B cells were verified by PCR amplification with primers outside of the *Setx* homology donor sequences and Bam HI digestion (Bam HI recognition sequences reside in the 3HA coding sequence) of the resulting amplicons. PCR amplicons from cells with both *Setx* alleles successfully targeted could be completely digested by Bam HI, while amplicons from the WT allele remained resistant to Bam HI.

Abl pre-B cell lines carrying missense mutations K1945R (*Setx^K1945R/K1945R^*) and L2131W (*Setx^L2131W/L2131W^*) were generated by targeted integration of the recombination donor DNA constructs containing the indicated mutations and 800-bp *Setx* genomic sequences 5′ and 3′ of the mutations in *Setx^3HA/3HA^* abl pre-B cells as described above. Synonymous mutations were incorporated in the targeting constructs to create recognition sequences for Eco NI (K1945R targeting construct) and Xho I (L2131W targeting construct). Biallelically targeted *Setx^K1945R/K1945R^* and *Setx^L2131W/L2131W^* mutant clones were verified by digesting PCR amplicons covering the respective mutations using primers outside of the donor sequences with Eco NI and Xho I, respectively, as described above.

All cell lines were grown in Dulbecco’s modified Eagle’s medium (no l-glutamine, Gibco, 11960077) supplemented with 10% heat-inactivated fetal bovine serum (Gibco, A52567-1), penicillin/streptomycin (100 U/ml; Gibco, 15140122), 1 mM sodium pyruvate (Gibco, 11360070), 2 mM l-glutamine (Gibco, 25030081), 1X non-essential amino acids (Gibco, 11-140-050), and 55 μM beta-mercaptoethanol (Sigma-Aldrich, M3148_100ML) at 37°C. Imatinib (S2475, used at 3 μM), the ATM kinase inhibitor KU55933 (S1092, used at 15 μM), and the DNA-PKcs kinase inhibitor NU7441 (S2638, used at 5 μM) were from Selleckchem. The CDK7 inhibitor THZ1 (9002615) was from Cayman Chemical.

### Genome-wide CRISPR-Cas9 screens

Four abl pre-B cell lines with integrated tetracycline-inducible Cas9 transgenes were used in genome-wide CRISPR-Cas9 gRNA screens: WT (M63.1: iCas9.302: pMG-INV 36), *Setx*^−/−^. 38 [derived from WT (M63.1: iCas9.302: pMG-INV 36)], *B. Setx*^−/−^.10 (derived from B. WT), and *Setx^−/−^: Recql5^−/−^. 5* (derived from *Setx*^−/−^. 38). For each screen, ~180 × 10^6^ cells were transduced with a lentiviral gRNA library containing 90,230 gRNAs targeting 18,424 mouse genes (Addgene, #67988) at a 40 to 50% transduction efficiency, as determined by the percentage of blue fluorescent protein (BFP)–positive cells after transduction. Three days after transduction, stably transduced cells expressing BFP were isolated by FACS and treated with doxycycline (2 μg/ml) for 6 days to induce Cas9 expression and genome-wide gene inactivation. Cells were then treated with 3 μM imatinib for 4 days as described above to induce V(D)J recombination of pMG-INV. In the screen using WT (M63.1: iCas9.302: pMG-INV 36) abl pre-B cells, one set of the cells was also exposed to 5 μM NU7441 during imatinib treatment. Cells that had (GFP^+^) and had not (GFP^−^) undergone pMG-INV recombination were purified by FACS, and genomic DNA was isolated from these cells. The gRNAs from each population were PCR-amplified and sequenced on an Illumina NextSeq500 platform at the Genomic Core Laboratory of Heflin Center for Genomic Sciences at University of Alabama at Birmingham (UAB). Primers used in the PCR reactions are listed in table S8.

The gRNA sequences were retrieved from FASTQ files using Seqkit (*73*). The derived sequences were mapped to the reference gRNA sequences of the library (*74*). The number of reads of each gRNA were normalized as follows: Normalized reads of a particular gRNA = $\frac{\text{Reads of}\;a\;\text{gRNA}}{\text{Total reads for}\;\text{all}\;\text{gRNAs in}\;a\;\text{sample}}$ × 10^6^ + 1] (*75*). The enrichment score of a gRNA is calculated as the ratio of normalized reads of the gRNA between two samples (GFP^−^ versus GFP^+^).

### Flow cytometric analysis

Flow cytometric analysis of GFP expression of imatinib-treated abl pre-B cells and bromodeoxyuridine (BrdU)/7-amino-actinomycin D (7AAD) staining for cell cycle profiling was conducted on LSRFortessa X-20 analyzers (BD Biosciences) supported by the Flow Cytometry and Single Cell Core and Stem Cell Shared Facility of Division of Hematology and Oncology, UAB. The data analyzed was performed using FlowJo software (FlowJo, LLC). The flow cytometry data, including multiple time points and at least three independent assays for each experiment, was summarized in table S3. *P* values were calculated by two-tailed Student’s *t* test.

### Southern blot analysis

To analyze V(D)J recombination products and intermediates of pMX-DEL^CJ^ and pMX-DEL^SJ^ by Southern blot, these retroviral recombination substrates were bulk-transduced to the abl pre-B cells indicated, and the stably transduced cells were isolated by the expression of the human CD4 (hCD4) surface protein marker by MACS cell separators and MS columns (Miltenyi Biotec). We note that the pMX-DEL^CJ^ and pMX-DEL^SJ^ recombination substrates were transduced to the indicated abl pre-B cells for each individual experiments without further screening for single integration or recombination efficiency. Therefore, multiple copies of the recombination substrates in different genomic locations may be present in the cells analyzed and result in varied and reduced RAG cleavage efficiency due to the inclusion of the substrates becoming inaccessible to RAG after integration (fig. S19) (*20*, *24*). Genomic DNA (8 μg) purified from untreated or imatinib-treated abl pre-B cells with pMX-DEL^CJ^ or pMX-DEL^SJ^ was digested with Eco RV and resolved in 1.2% tris-acetate-EDTA gels. Upon transferring DNA to Whatman Nytran SPC membranes (Cytiva, 10416296), restriction fragments corresponding unrearranged reporter (UR), CJ, CE, signaling joint (SJ), and SE were visualized using the hCD4 gene fragment as the probe template as previously described (*24*). The same blots were stripped off the hCD4 probe and reprobed with a β T cell receptor probe for loading control. The Thy1 cDNA was used as the probe template for Southern blot analysis of the pMG-INV substrate on Nhe I– or Xba I–digested genomic DNA. For monitoring the repair of Cas9 DSBs at the enhancer beta region (Eb) of the endogenous *Tcrb* locus, Hind III–digested genomic DNA was probed with an Eb DNA sequence.

### Western blot analysis

For Western blot analysis, protein samples were resolved in 4 to 12% NuPAGE bis-tris gels or 3 to 8% NuPAGE gels followed by wet transfer to 0.45-μm Protran nitrocellulose membranes (Cytiva, 10600002). The membranes were blocked in 5% non-fat milk for 30 min at room temperature and probed with antibodies as indicated. Antibodies to RECQL5 (12468-2-AP) and MRE11 (10744-1-AP) are from Proteintech. HA antibody (901501) is from BioLegend. DNA-PKcs antibody is from Thermo Fisher Scientific (MS-423-P). HLTF antibody is from Bethyl Laboratories (A300-230A). glyceraldehyde-3-phosphate dehydrogenase antibody is from Sigma-Aldrich (G8795). KAP1 antibody is from Cell Signaling Technology (4123).

### Radiosensitivity assay

The abl pre-B cells (3 to 4 × 10^6^) treated with imatinib for 2 days were seeded in each well of 12-well plates in triplicate and irradiated using X-RAD320 x-ray irradiator. Two days after irradiation, 450 μl of treated or untreated cells was mixed with 50 μl of PrestoBlue HS reagent (Thermo Fisher Scientific) for 6 hours at 37°C. The absorbances of the resulting PrestoBlue HS media mixture were measured at 560 and 590 nm on a Synergy H1 Hybrid Reader (BioTek) microplate spectrophotometer, in accordance with the manufacturer’s instructions.

### Analysis of structural variants of SJs from pMG-INV by next-generation sequencing

To determine structural variants of SJs, genomic DNA from imatinib-treated abl pre-B cells was first subject to PCR to generate amplicons 1.1 kb in size with primers pMG-INV SJ F4 and pMG-INV 3′-3 R flanking the junction of SJs. The 1.1-kb amplicon was used as the template in an additional round of PCR with primers PE.P5_SJ23 and P7_SJ12 located 130 bp to the 5′ and 3′ of the SJ junction to generate a ~260-bp product with Illumina indexes and adaptors for 150-bp paired end sequencing using miSeq Micro platform (Illunima). Primers used in the PCR reactions are listed in table S8.

The sequencing results were analyzed, and structural variants were identified as previously described (*76*). Briefly, reads were demultiplexed based on their index sequences. After removal of adapter sequences, low-quality reads, and trimming reads that were shorter than 20 bp by using cutadapt (v1.3.1), filtered reads were aligned with bowtie2 (version 2.1.0) to the 295-bp pMG-INV SJ reference sequence (data S1). With mapped reads in BAM format as input, structural variants were called by using Pindel (*77*). Indels and their microhomology status that span the break point site (the 165-nt position in the target region) with ≥5 supporting reads were extracted, counted, summarized, and compared among different conditions (table S7). *P* values (table S7) were calculated by two-tailed Student’s *t* test.
