# Supplementary material for: Senataxin and DNA-PKcs redundantly promote non-homologous end joining repair of DNA double strand breaks during V(D)J recombination
Source: Sci Adv. 2025 Jun 20;11(25):eads5272. doi: 10.1126/sciadv.ads5272 (PMC12180482; doi:10.1126/sciadv.ads5272)
Supplement: Supplementary file 1 — Figs. S1 to S19 Tables S2, S3 and S8 Data S1 Legends for tables S1, S4 to S7 [file sciadv.ads5272_sm.pdf]

Science Advances
AAAS

Supplementary Materials for

Senataxin and DNA-PKcs redundantly promote non-homologous end joining repair of DNA double strand breaks during V(D)J recombination

Bo-Ruei Chen *et al.*

Corresponding author: Bo-Ruei Chen, bchen@uabmc.edu; Barry P. Sleckman, bps@uab.edu

Sci. Adv. **11**, eads5272 (2025)
DOI: 10.1126/sciadv.ads5272

The PDF file includes:

Figs. S1 to S19
Tables S2, S3 and S8
Data S1
Legends for tables S1, S4 to S7

Other Supplementary Material for this manuscript includes the following:

Tables S1, S4 to S7

Supplementary Figure S1

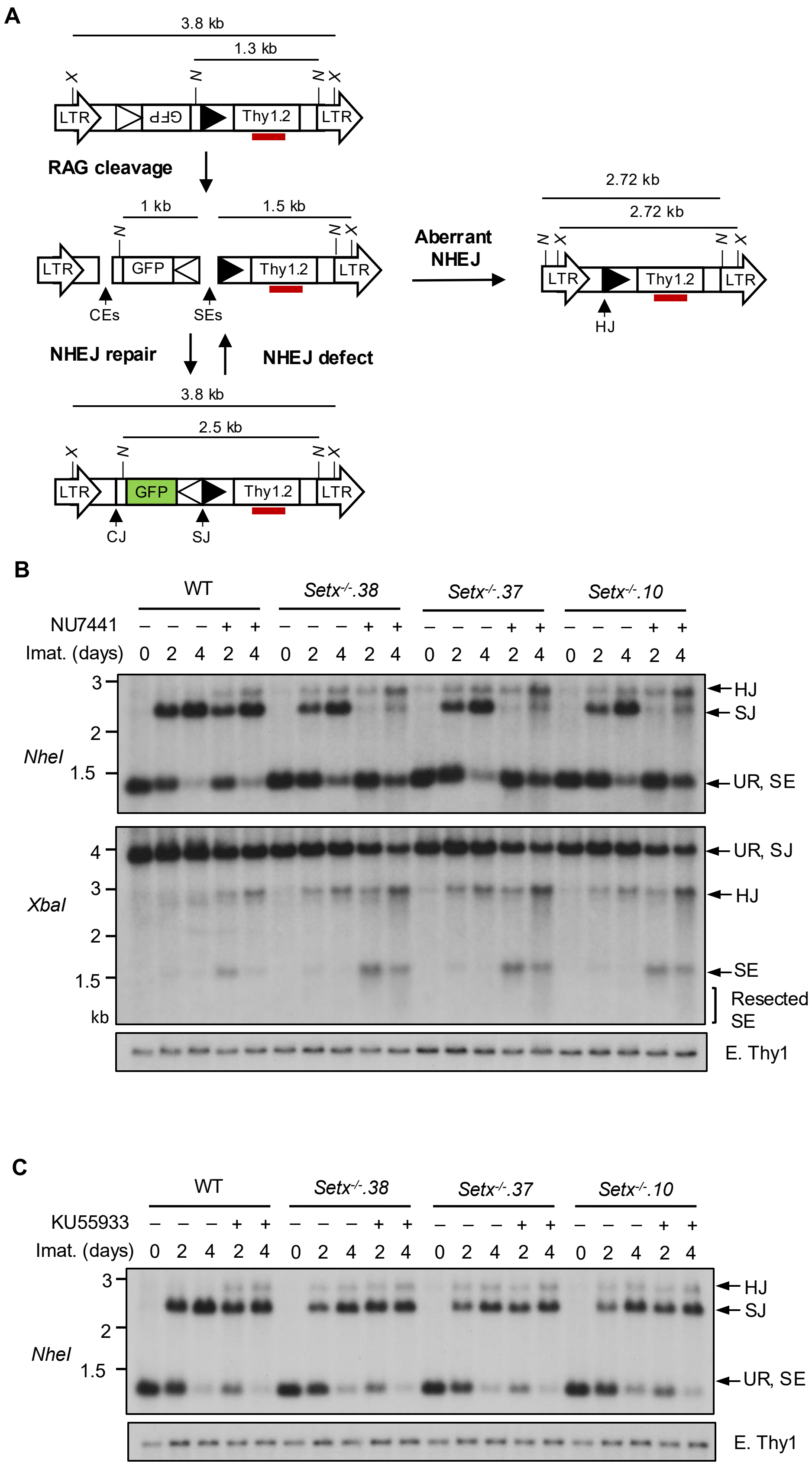

Figure S1. Senataxin is differentially required for NHEJ-mediated RAG DSB repair in response to inhibition of DNA-PKcs and ATM. (A) The schematic of the retroviral inversional V(D)J recombination substrate pMG-INV. The open and filled triangles represent the RSSs. The red bar indicates the probe (Thy1 cDNA) for Southern blot. The Xs and Ns denote the *Xba*I *and Nhe*I restriction sequences. The retroviral LTRs, CEs and SEs upon RAG cleavage as well as CJs and SJs after NHEJ-mediated repair are indicated. Aberrant joining of a CE and a SE leading to a HJ is also shown. (B) Southern blot analysis of genomic DNA from WT and three clonal *Setx*$^{-/-}$ abl pre-B cell lines with pMG-INV treated with imatinib (imat.) in the presence or absence of the DNA-PKcs kinase inhibitor NU7441 for the indicated times. The genomic DNA samples were digested with *Nhe*I (top) *or Xba*I (bottom) and hybridized with the Thy1 probe. The restriction fragments corresponding to unrearranged reporters (UR), repaired SJs or HJs and unrepaired SEs are indicated. E. Thy1: Endogenous Thy1 restriction fragment, loading control. (C) Southern blot of *Nhe*I-digested genomic DNA from abl pre-B cells as in *(B)* but treated with imatinib +/- the ATM inhibitor KU55933 for the indicated times.

Supplementary Figure S2

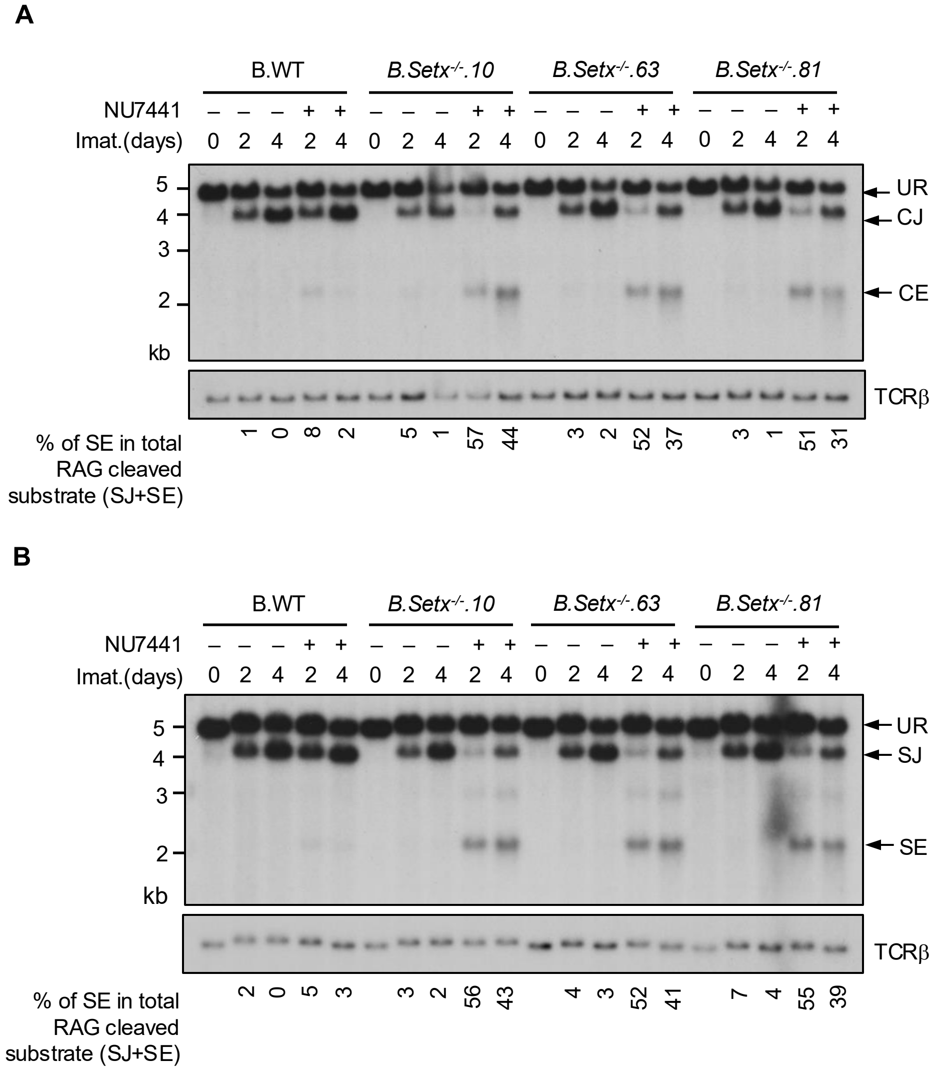

Figure S2. Loss of senataxin severely impairs NHEJ-mediated RAG DSB repair in DNA-PKcs-inhibited abl pre-B cells during V(D)J recombination. Southern blot analysis of *EcoR*V-digested genomic DNA from additional WT (B. WT) and its derived clonal *Setx*$^{-/-}$ abl pre-B cell lines with pMX-DEL^{CJ} *(A)* or pMX-DEL^{SJ} *(B)* treated with imatinib (imat.) in the presence or absence of the DNA-PKcs kinase inhibitor NU7441 for the indicated times.

Supplementary Figure S3

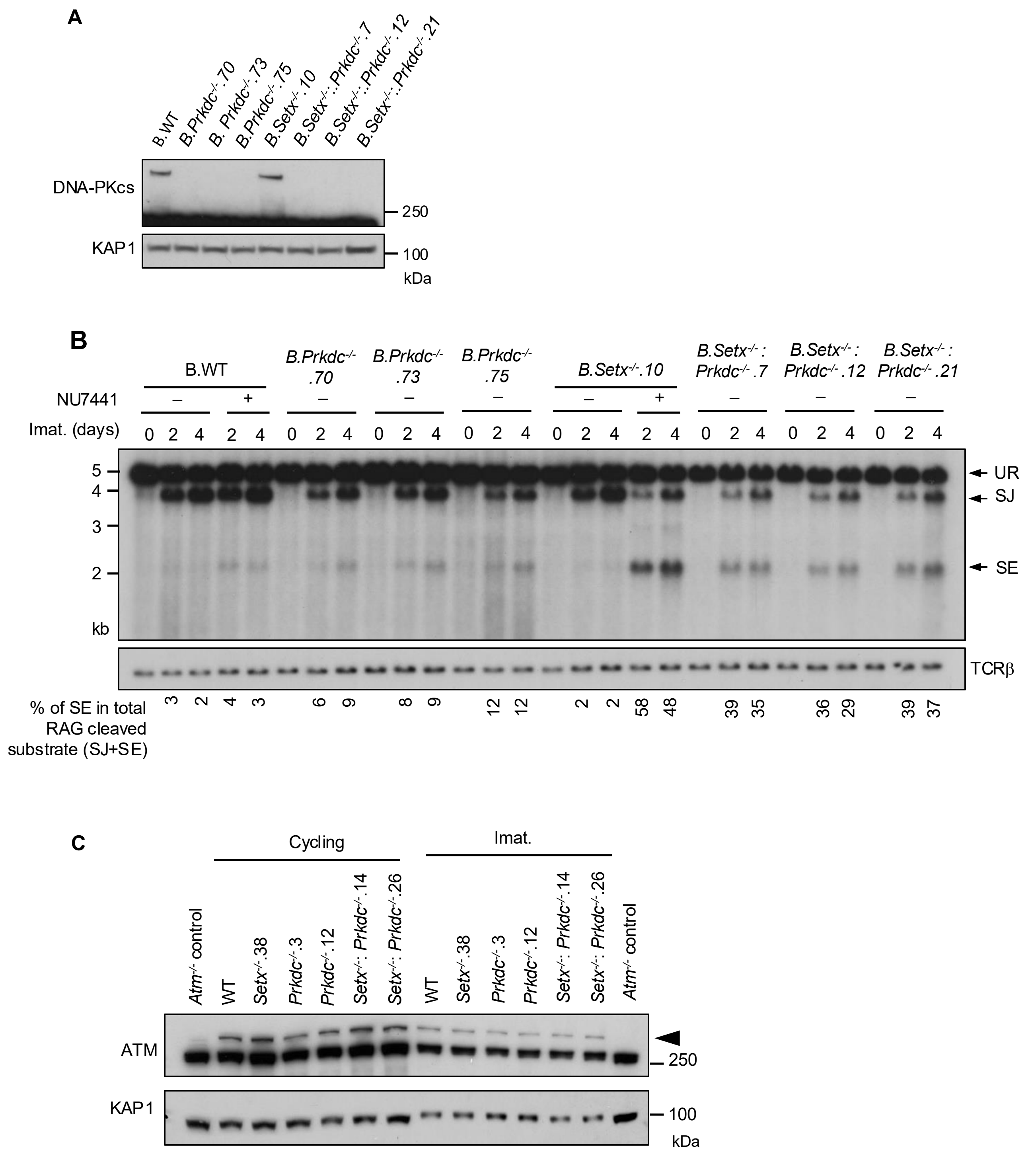

Figure S3. Combined loss of senataxin and DNA-PKcs proteins impairs NHEJ-mediated RAG DSB repair. (A) Western blot of cell lysates from additional WT (B. WT) and its derived *Prkdc*$^{-/-}$, *Setx*$^{-/-}$ and *Setx*$^{-/-}$: *Prkdc*$^{-/-}$ abl pre-B cell lines using DNA-PKcs and KAP1 antibodies. (B) Southern blot analysis of *EcoR*V-digested genomic DNA isolated from WT, *Prkdc*$^{-/-}$, *Setx*$^{-/-}$ and *Setx*$^{-/-}$: *Prkdc*$^{-/-}$ abl pre-B cells pMX-DE^{SJ} and treated with imatinib (imat.) in the presence or absence of the DNA-PKcs inhibitor NU7441 for the indicated times. (C) Western blot of cell lysates from WT, *Prkdc*$^{-/-}$, *Setx*$^{-/-}$ and *Setx*$^{-/-}$: *Prkdc*$^{-/-}$ abl pre-B cell lines in cycling or after imatinib treatment using ATM and KAP1 antibodies.

Supplementary Figure S4

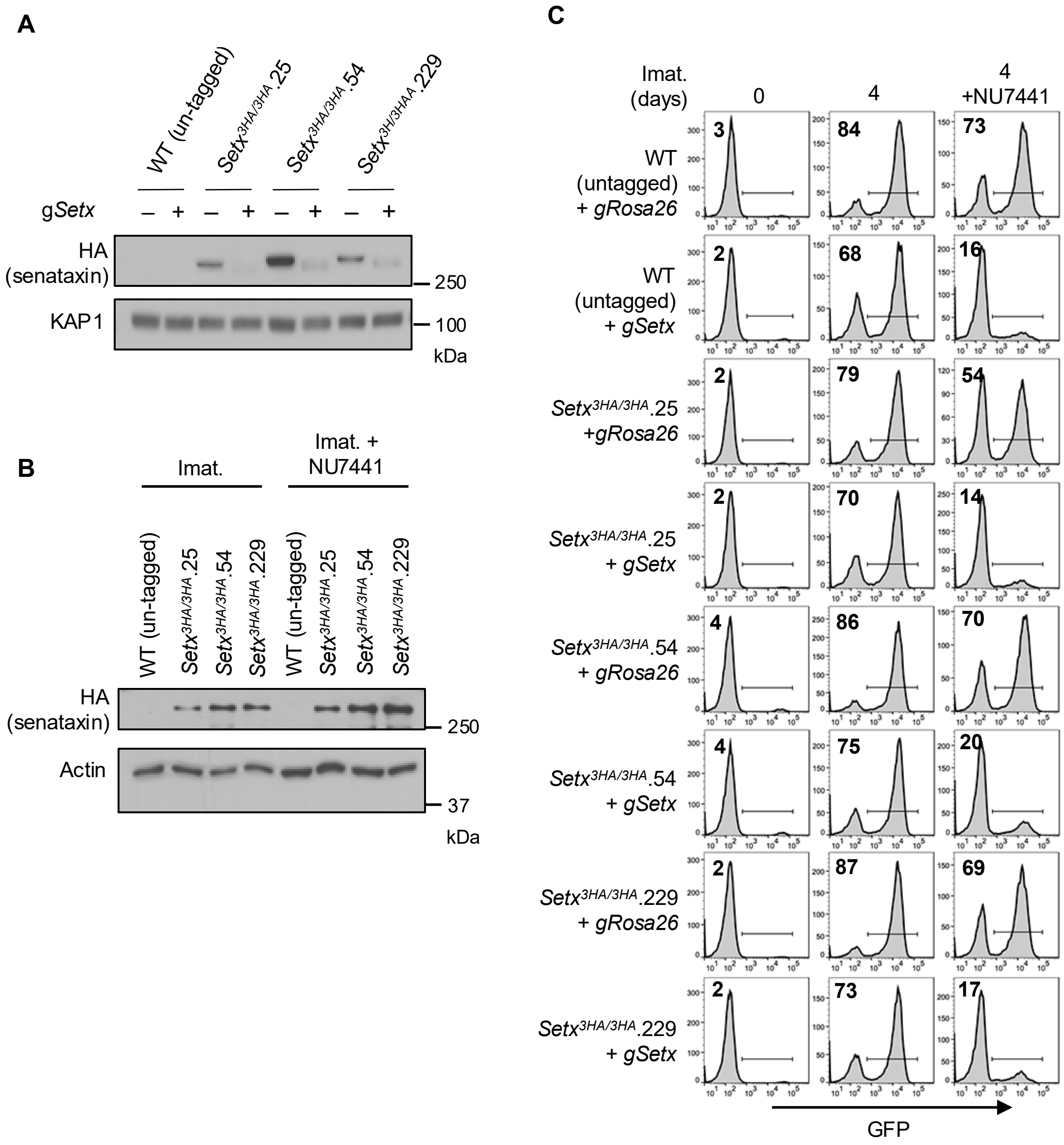

Figure S4. Characterization of abl pre-B cells expressing endogenous senataxin with a C-terminally tagged triple HA epitope (3HA). (A) Western blot analysis of cell lysates from proliferating WT and *Setx*$^{3HA/3HA}$ abl pre-B cells expressing Cas9 with or without Setx gRNAs (*gSetx*) using HA and KAP1 antibodies. (B) Western blot analysis of cell lysates from imatinib (imat.)-treated WT and *Setx*$^{3HA/3HA}$ abl pre-B cells with or without the DNA-PKcs kinase inhibitor NU7441 for 2 days using HA and actin antibodies. (C) Flow cytometric analysis for GFP expression from pMG-INV in WT and *Setx*$^{3HA/3HA}$ abl pre-B cells expressing Cas9 and *gRosa26* or *gSetx*, treated with imatinib in the presence or absence of NU7441 for the indicated times. The percentages of GFP^{+} cells are indicated in the top left corners of the histograms.

Supplementary Figure S5

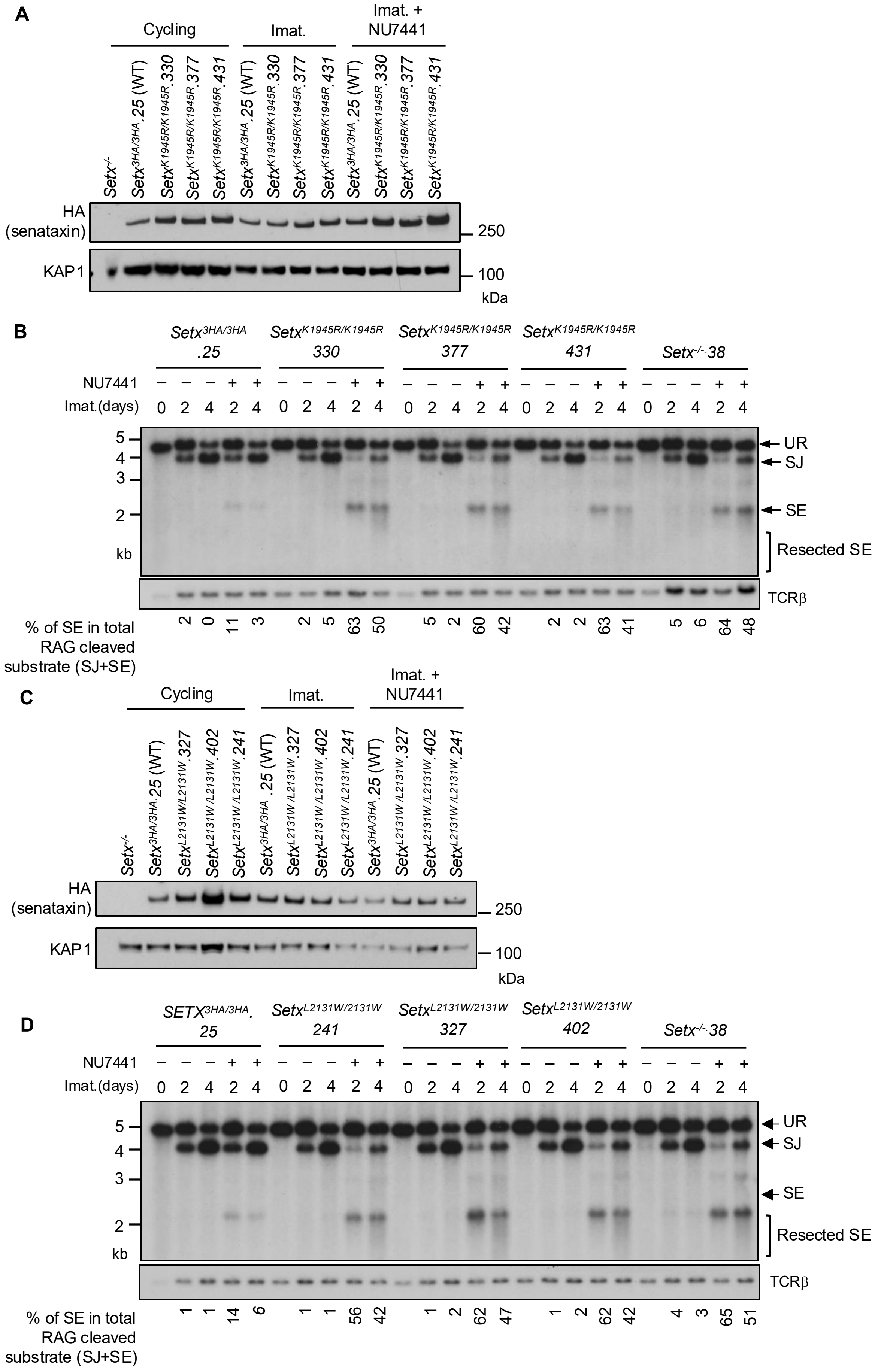

Figure S5. The K1945R and L2131W mutations of senataxin impair its NHEJ function. (A, C) Western blot analysis of cell lysates from *Setx*$^{-/-}$, *Setx*$^{3HA/3HA}$ and three clones of *Setx*$^{K1945R/K1945R}$ (*A*) or *Setx*$^{L2131W/L2131W}$ (*C*) abl pre-B cells in proliferation (cycling) or after treatment with imatinib (imat.) in the presence of absence of DNA-PKcs kinase inhibitor NU7441 for 2 days using HA and KAP1 antibodies. (B, D) Southern blot analysis of *EcoR*V-digested genomic DNA from *Setx*$^{K1945R/K1945R}$ (*B*) or *Setx*$^{L2131W/L2131W}$ (*D*) abl pre-B cells with pMX-DEL^{SJ} after imatinib treatment with or without NU7441 for the indicated times.

Supplementary Figure S6

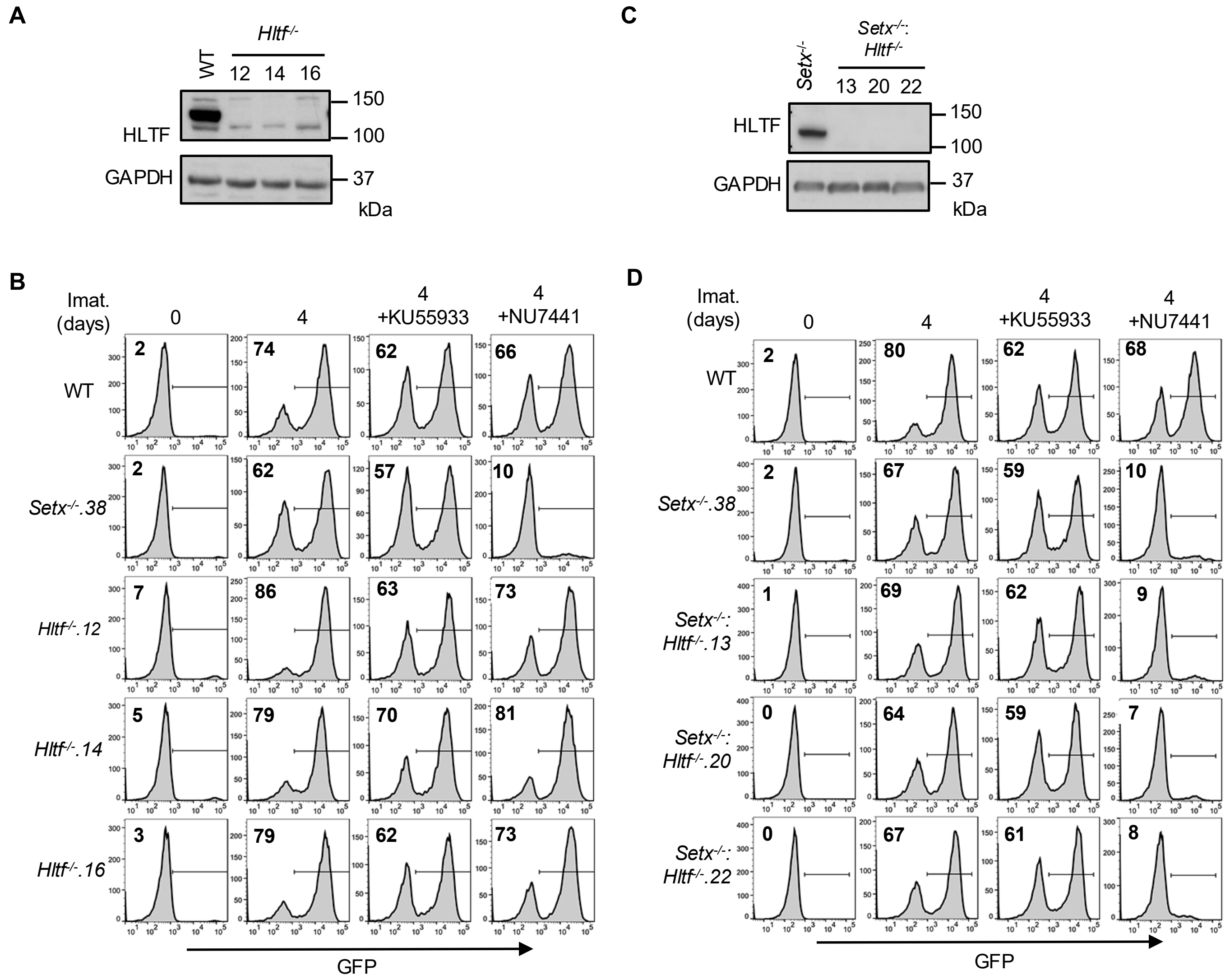

Figure S6. *Hltf*$^{-/-}$ and *Setx*$^{-/-}$: *Hltf*$^{-/-}$ abl pre-B cells exhibit no discernible defect in V(D)J recombination. (A) Western blot analysis of cell lysates from WT and three independently isolated *Hltf*$^{-/-}$ abl pre-B cell lines using HLTF and GAPDH antibodies. (B) Flow cytometric analysis of GFP expression from pMG-INV in WT, *Setx*$^{-/-}$ and three clones of *Hltf*$^{-/-}$ abl pre-B cells treated with imatinib alone or in the presence of the ATM kinase inhibitor KU55933 or DNA-PKcs kinase inhibitor NU7441 for the indicated times. The percentages of GFP^{+} cells are shown in the top left corners of the histograms. (C) Western blot analysis of cell lysates from *Setx*$^{-/-}$ and three independently isolated *Setx*$^{-/-}$: *Hltf*$^{-/-}$ abl pre-B cells using HLTF and GAPDH antibodies. (D) Flow cytometric analysis of GFP expression from pMG-INV in WT, *Setx*$^{-/-}$ and three clones of *Setx*$^{-/-}$: *Hltf*$^{-/-}$ abl pre-B cells as indicated in *(B)*.

Supplementary Figure S7

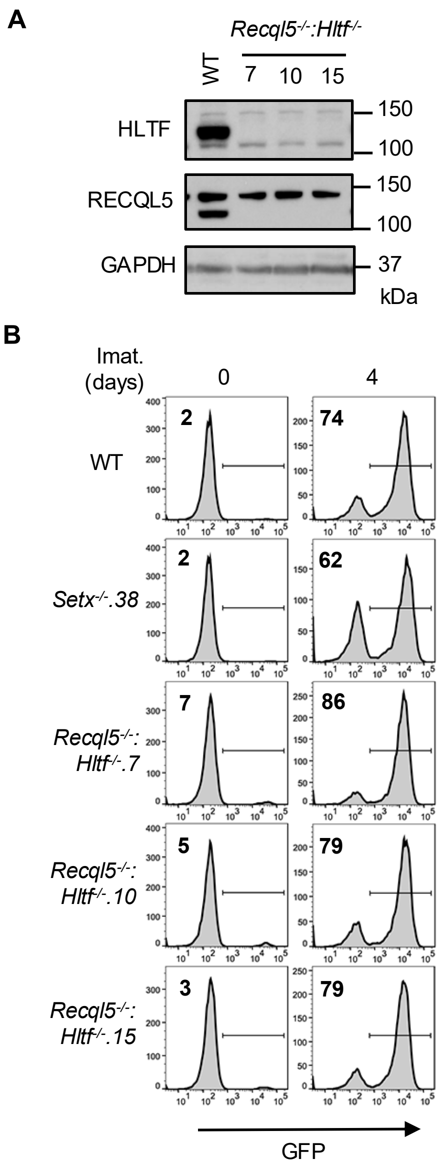

Figure S7: *Recql5*$^{-/-}$*: Hltf*$^{-/-}$ abl pre-B cells exhibit normal V(D)J recombination. (A) Western blot analysis of cell lysates from WT and three independently isolated *Recql5*$^{-/-}$: *Hltf*$^{-/-}$ abl pre-B cells using HLTF, RECQL5 and GAPDH antibodies. (B) Flow cytometric analysis of GFP expression from pMG-INV in WT, *Setx*$^{-/-}$ and three clones of *Recql5*$^{-/-}$: *Hltf*$^{-/-}$ abl pre-B treated with imatinib (imat.) for the indicated times. The percentages of GFP^{+} cells are indicated in the top left corners of the histograms.

Supplementary Figure S8

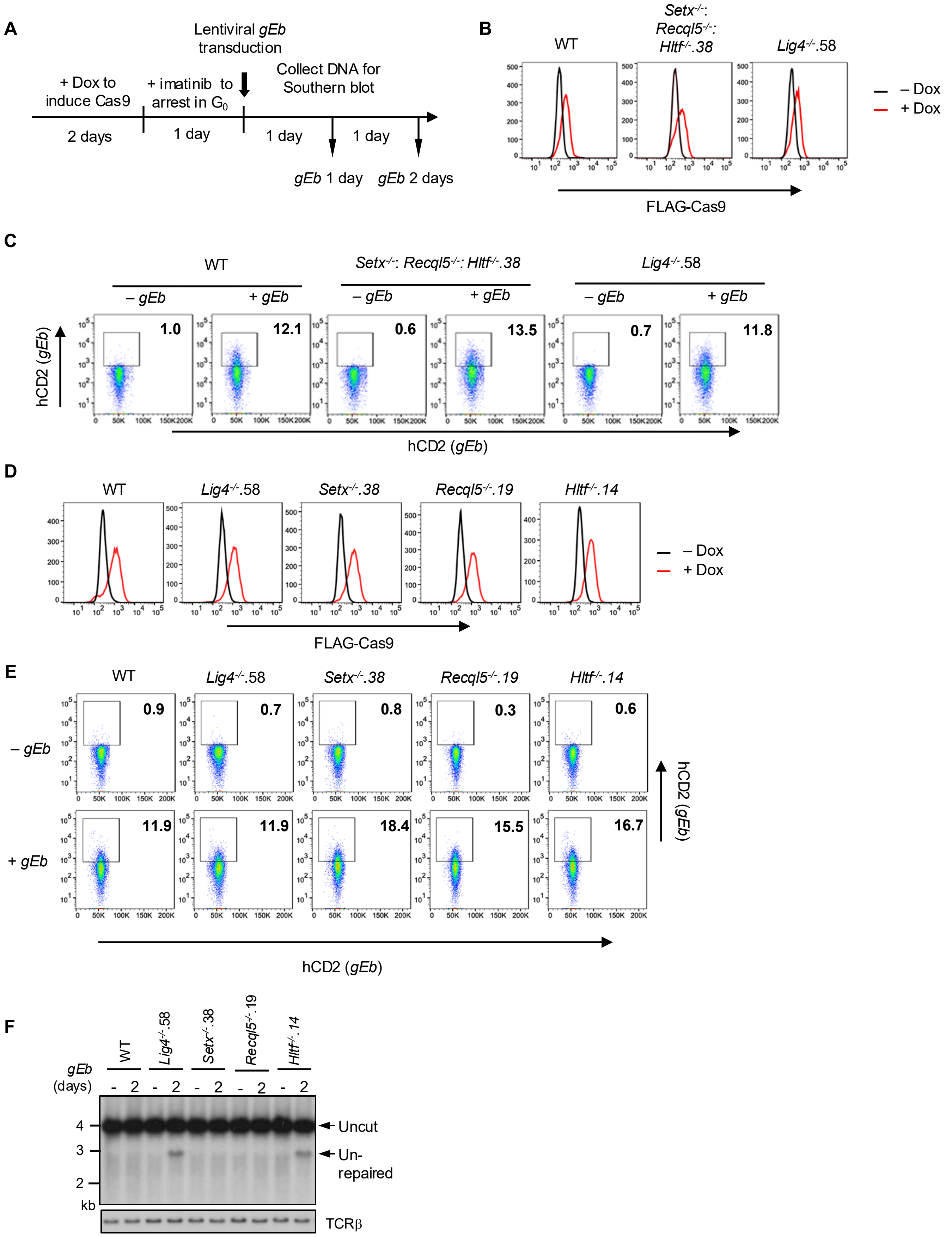

Figure S8: Analysis of Cas9 DSB repair in imatinib-treated, G_0-arrested abl pre-B cells. (A) The schematic of the experimental procedure for inducing Cas9 DSBs with *gEb* in imatinib-treated abl pre-B cells. (B, D) Flow cytometric analysis of 3XFLAG-Cas9 expression after doxycycline treatment in the indicated abl pre-B cells. (C, E) Flow cytometric analysis of the expression of hCD2, a marker on the lentiviral *gEb*-expressing pKLV-*gEb*-hCD2 vector 2 days after viral transduction. (F) Southern blot analysis of *Hind*III digested genomic DNA from the indicated abl pre-B cells after doxycycline and imatinib treatment to induce 3XFLAG-Cas9 and the Cas9 DSBs using the *Eb* probe.

Supplementary Figure S9

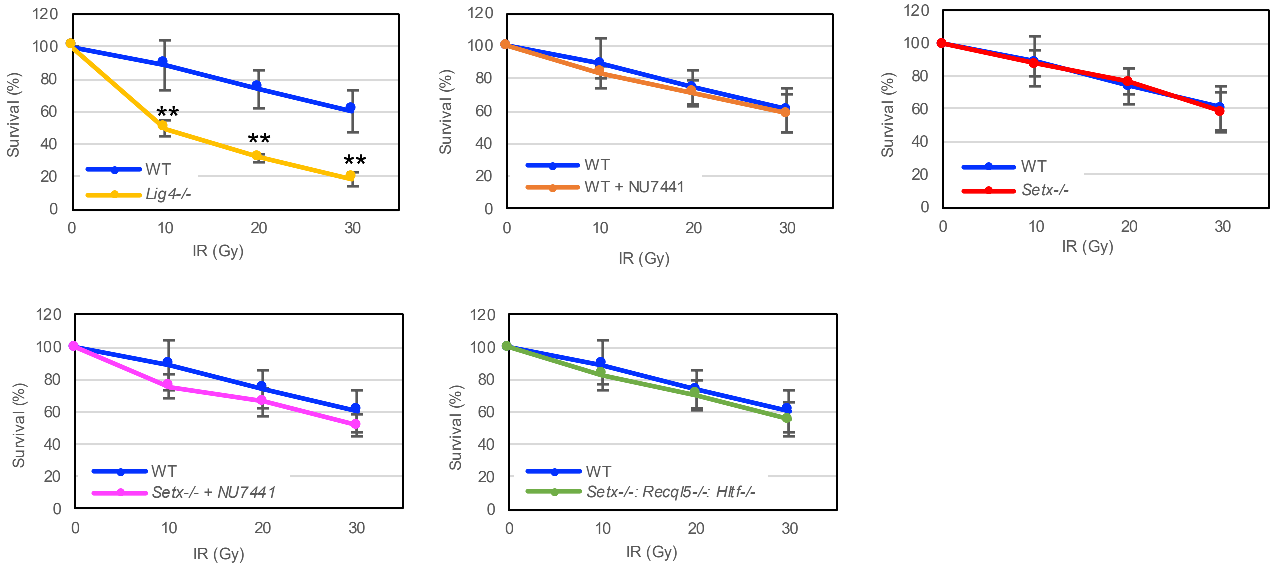

Figure S9: Sensitivity of imatinib-treated abl pre-B cells to ionizing radiation (IR). The viability of imatinib-treated abl pre-B cells of indicated genotypes, treated with or without the DNA-PKcs kinase inhibitor NU7441 as determined by the viability reagent PrestoBlue two days after irradiation. The same WT data were shown in all graphs to assist visualization and comparison of the results. The graphs show the average of 4 independent experiments. Error bars indicate standard deviations. Two-tailed Student's t-test was performed to determine significance. **: $P < 0.01$.

Supplementary Figure S10

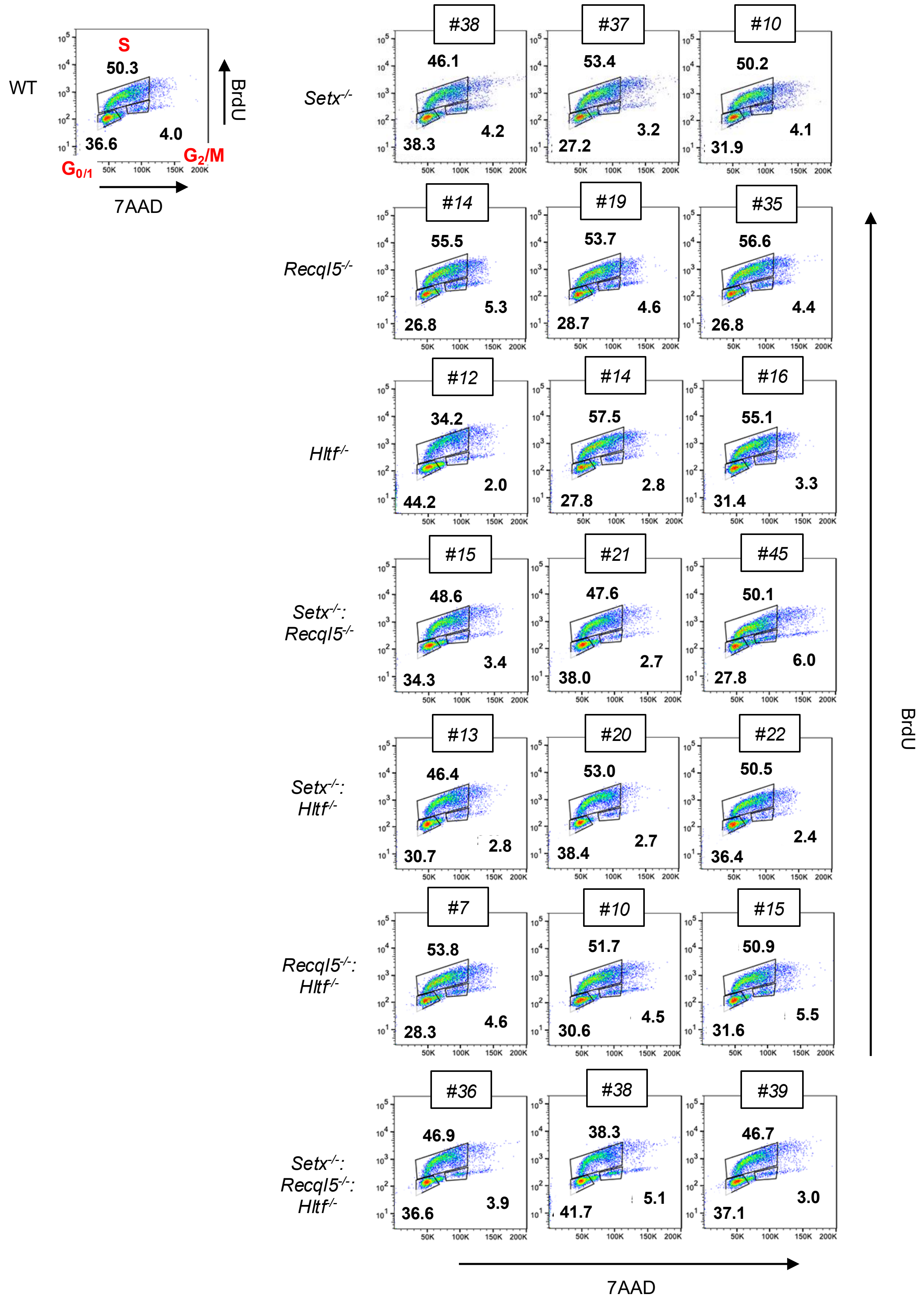

Figure S10. Cell cycle analysis of proliferating abl pre-B cells. Flow cytometric analysis of cell cycle distribution of proliferating abl pre-B cells of indicated genotypes after BrdU pulsing. The resulting cells were fixed and permeabilized before staining with APC-conjugated BrdU antibody and 7-AAD. The percentages of cells in G_1/G_0, S and G_2/M phases are shown in the dot plots.

Supplementary Figure S11

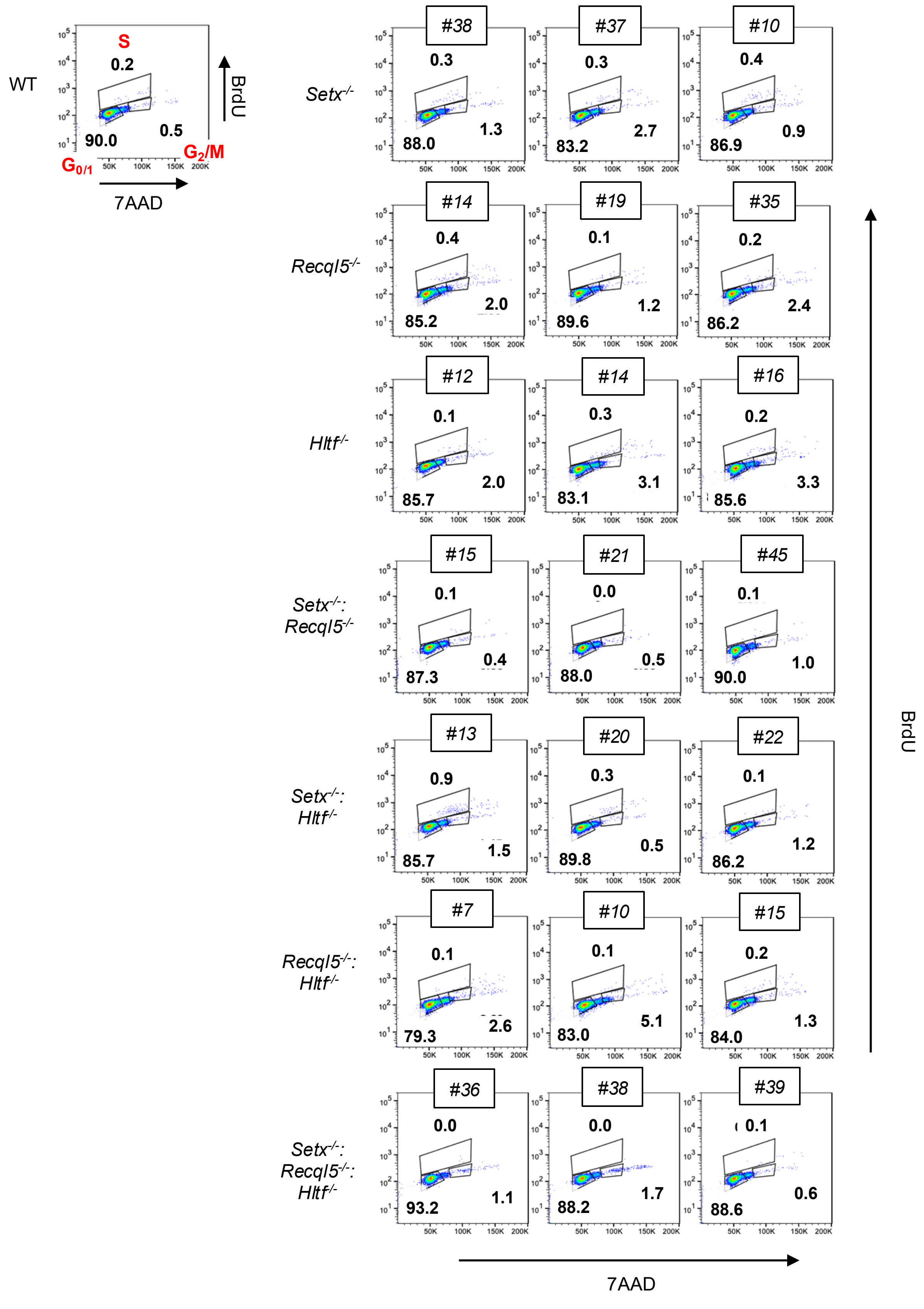

Figure S11. Cell cycle analysis of imatinib-treated abl pre-B cells. Flow cytometric analysis of cell cycle distribution of imatinib-treated (2 days) abl pre-B cells of indicated genotypes after BrdU pulsing. The resulting cells were fixed and permeabilized before staining with APC-conjugated BrdU antibody and 7-AAD. The percentages of cells in G_1/G_0, S and G_2/M phases are shown in the dot plots.

Supplementary Figure S12

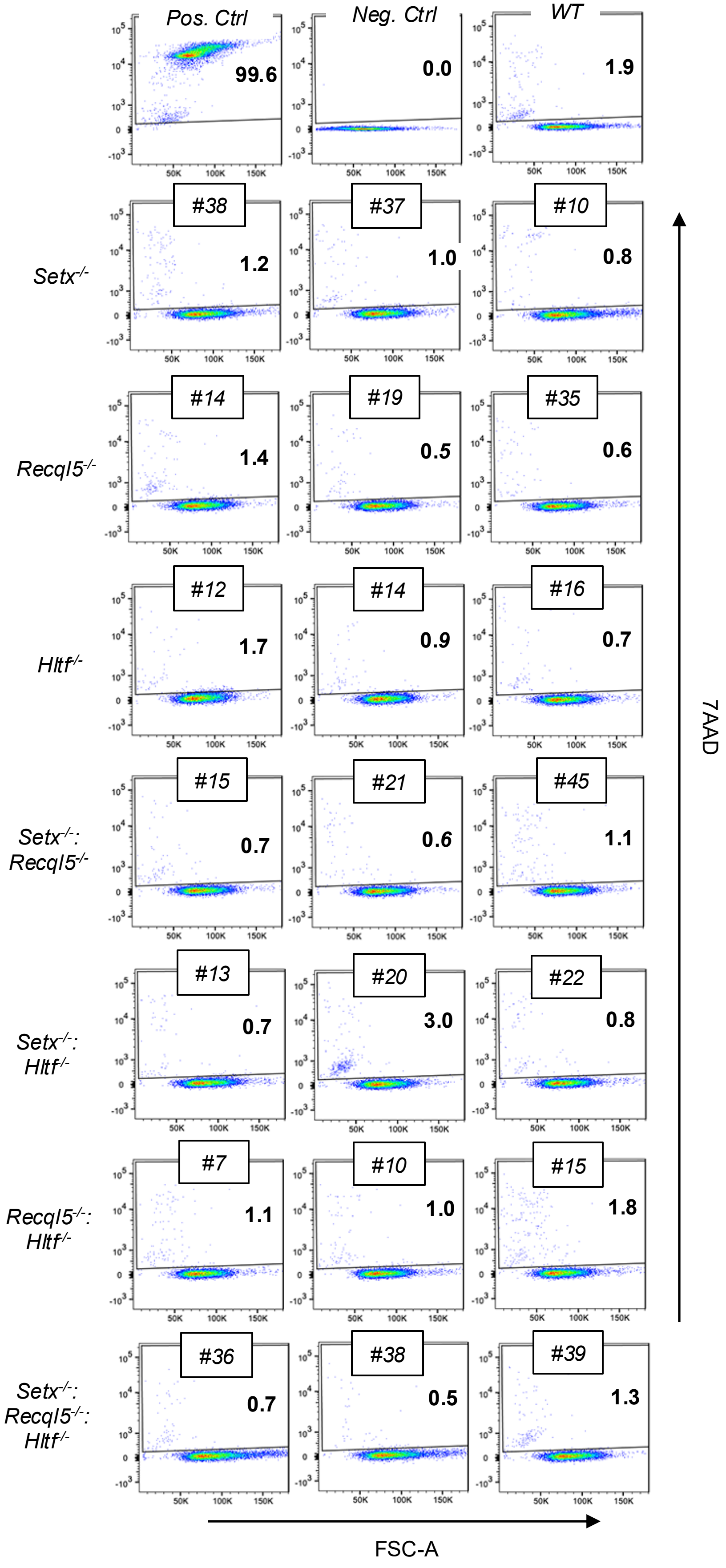

Figure S12. Cell viability analysis of proliferating abl pre-B cells. Flow cytometric analysis of 7AAD-stained proliferating abl pre-B cells of the indicated genotypes from live cell cultures. The positive control was from staining cells fixed and permeabilized with methanol and the negative control was from unstained live cells.

Supplementary Figure S13

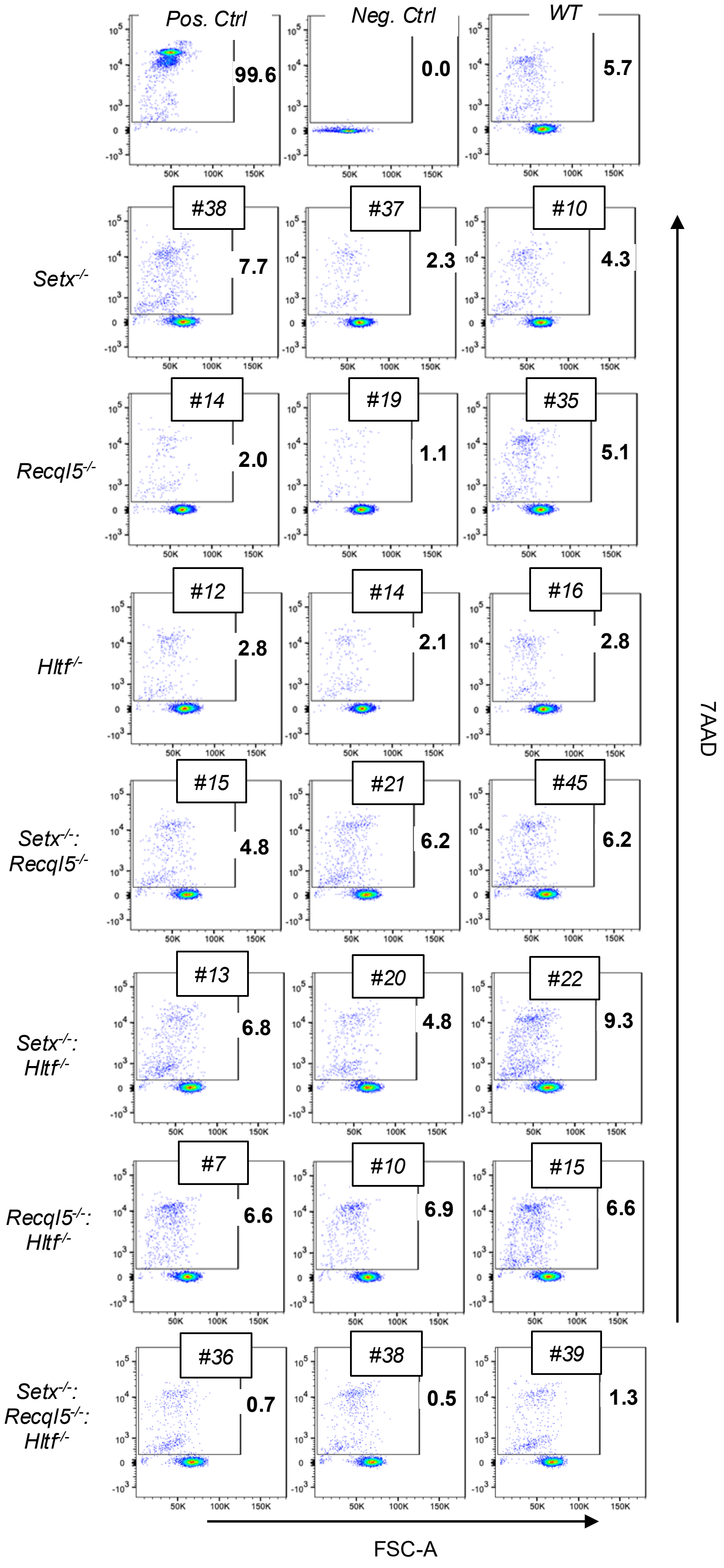

Figure S13. Cell viability analysis of imatinib-treated abl pre-B cells. Flow cytometric analysis of 7AAD-stained, imatinib-treated (2 days) abl pre-B cells of the indicated genotypes from live cell cultures. The positive control was from staining cells fixed and permeabilized with methanol and the negative control was from unstained live cells.

Supplementary Figure S14

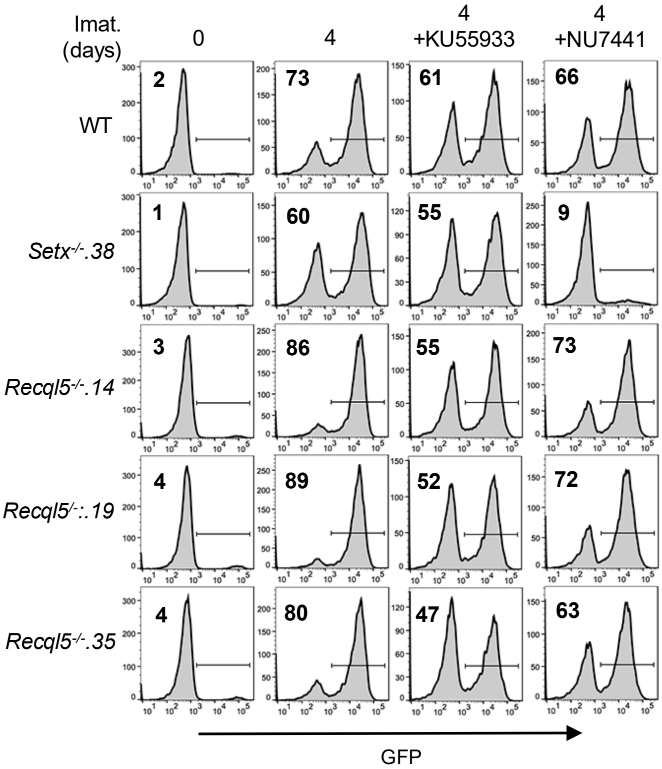

Figure S14. V(D)J recombination in *Recql5*$^{-/-}$ abl pre-B cells. Flow cytometric analysis of GFP expression from pMG-INV in WT, *Setx*$^{-/-}$ and three clones of *Recql5*$^{-/-}$ abl pre-B treated with imatinib (imat.) alone or in the presence of the ATM kinase inhibitor KU55933 and the DNA-PKcs kinase inhibitor NU7441 for the indicate times. The percentages of GFP^{+} cells are indicated in the top left corners of the histograms.

Supplementary Figure S15

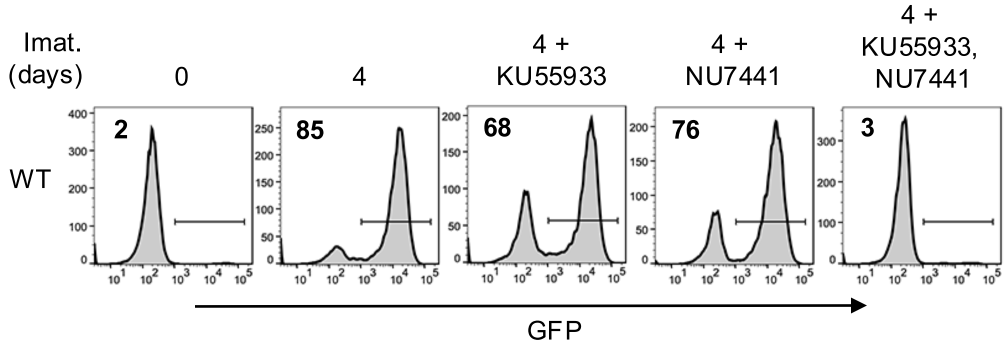

Figure S15. V(D)J recombination in WT abl pre-B cells with ATM and/or DNA-PKcs inhibition.

Flow cytometric analysis of GFP expression in imatinib (imat.)-treated WT abl pre-B cells +/- the ATM kinase inhibitor KU55933, the DNA-PKcs kinase inhibitor NU7441 or both for the indicated times.

Supplementary Figure S16

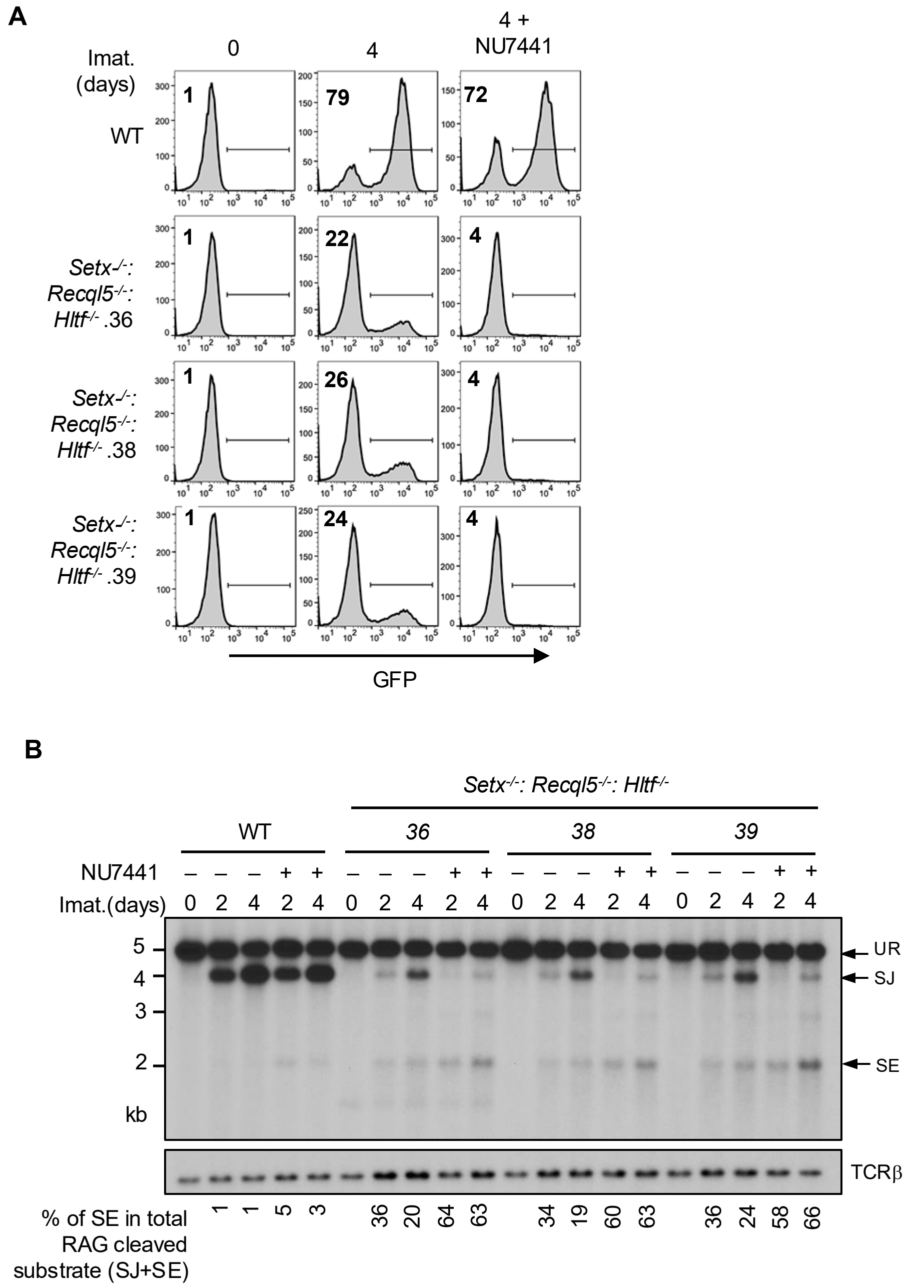

Figure S16. V(D)J recombination in *Setx*$^{-/-}$: *Recql5*$^{-/-}$: *Hltf*$^{-/-}$ with or without DNA-PKcs inhibition.

Flow cytometric analysis of GFP expression in imatinib (imat.)-treated WT or *Setx*$^{-/-}$: *Recql5*$^{-/-}$: *Hltf*$^{-/-}$ abl pre-B cells +/- the DNA-PKcs kinase inhibitor NU7441 for the indicated times.

Supplementary Figure S17

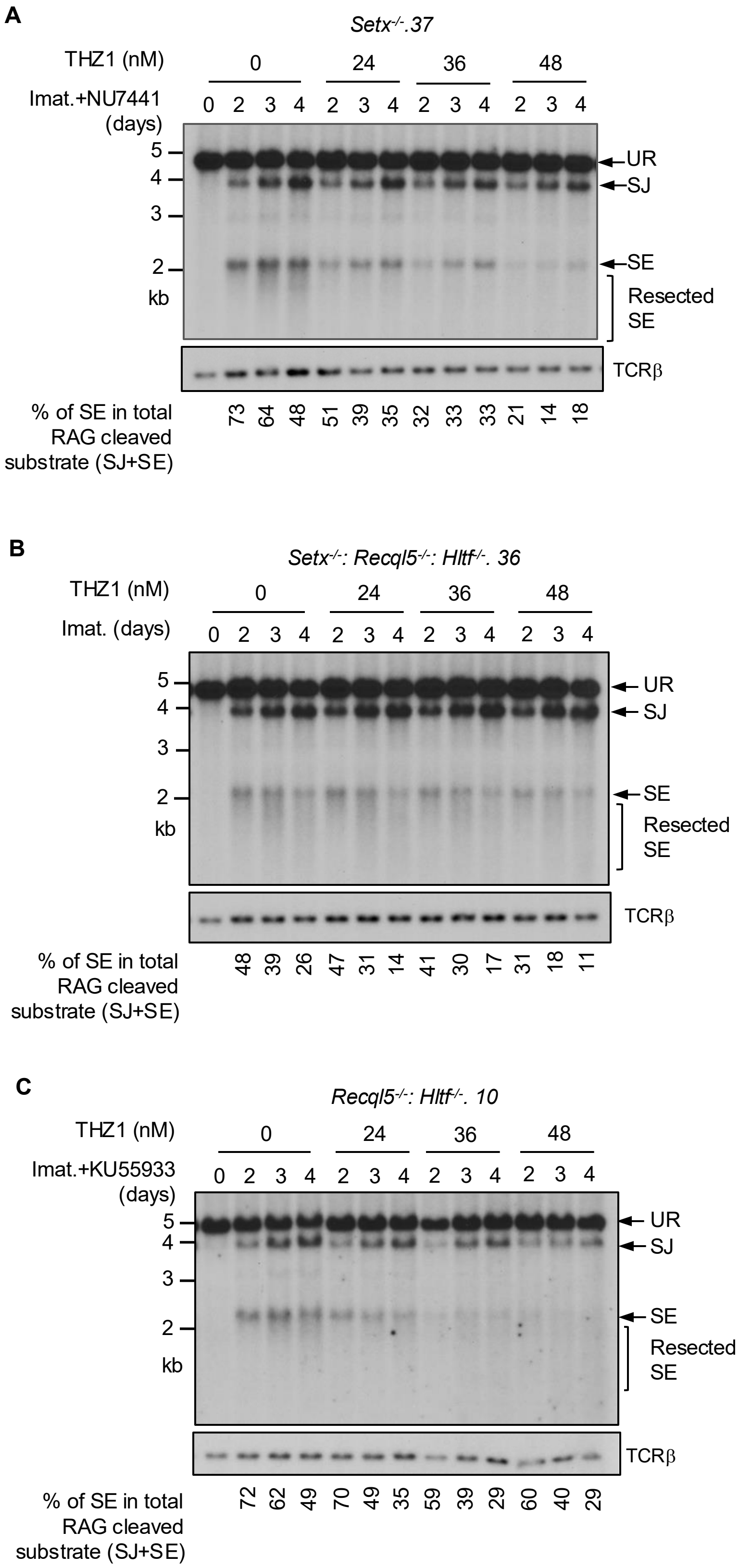

Figure S17. Partial inhibition of RNA polymerase II improves NHEJ-mediated RAG DSB repair in helicase-deficient abl pre-B cells. Southern blot analysis of *EcoR*V-digested genomic DNA from pMX-DEL^{SJ} containing, imatinib (imat.) treated *Setx*$^{-/-}$ (with the DNA-PKcs kinase inhibitor NU7441) (A), *Setx*$^{-/-}$: *Recql5*$^{-/-}$*: Hltf*$^{-/-}$ (B) and *Recql5*$^{-/-}$*: Hltf*$^{-/-}$ (with the ATM kinase inhibitor KU55933) (C) abl pre-B cells with increasing concentrations (24, 36 and 48nM) of the CDK7 inhibitor THZ1 for the indicated times.

Supplementary Figure S18

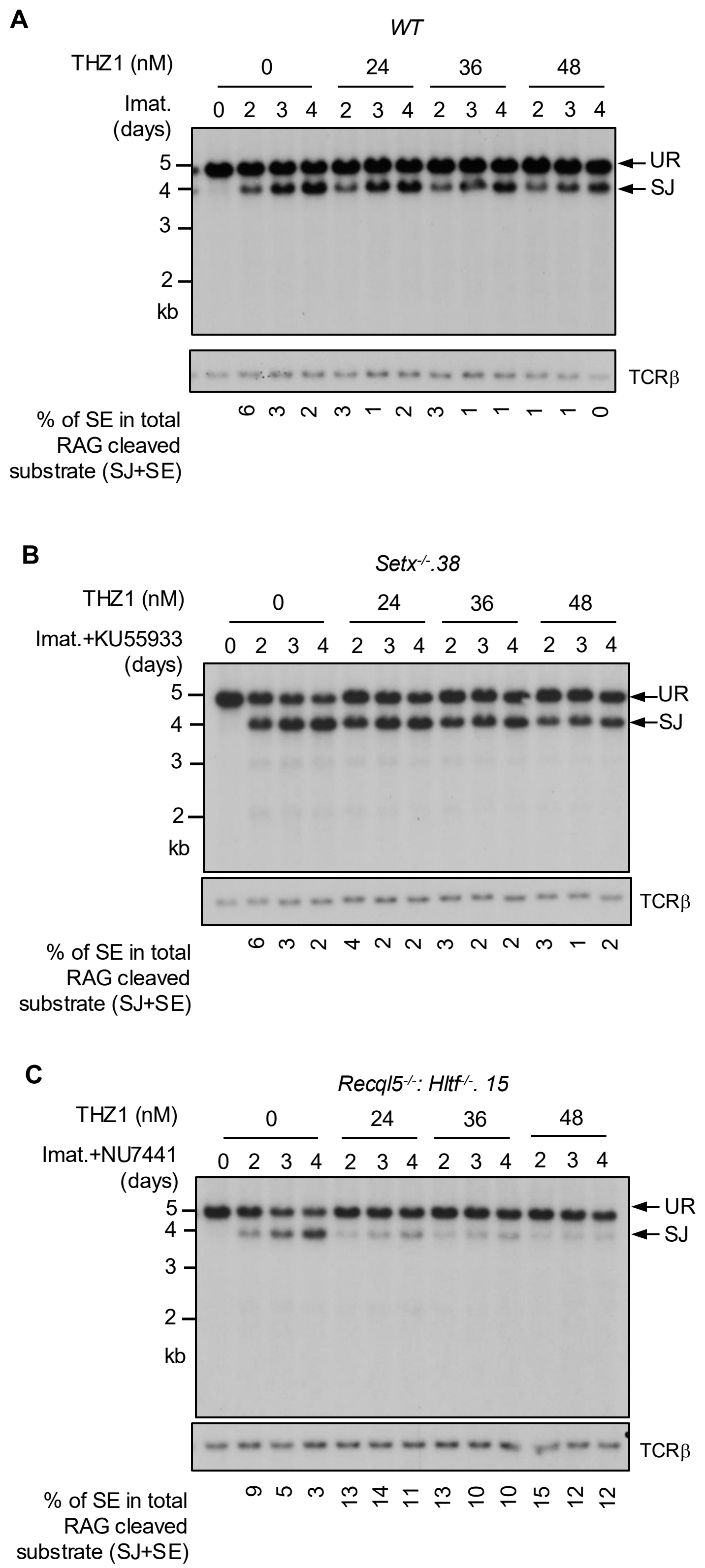

Figure S18. Partial inhibition of RNA polymerase II has no effect on RAG DSB repair in NHEJ-proficient abl pre-B cells. Southern blot analysis of *EcoR*V-digested genomic DNA from pMX-DEL^{SJ} containing, imatinib (imat.) treated WT (A), *Setx*$^{-/-}$ (with the ATM kinase inhibitor KU55933) (B) and *Recql5*$^{-/-}$*: Hltf*$^{-/-}$ (with the DNA-PKcs kinase inhibitor NU7441) (C) abl pre-B cells with increasing concentrations (24, 36 and 48nM) of the CDK7 inhibitor THZ1 for the indicated times.

Supplementary Figure S19

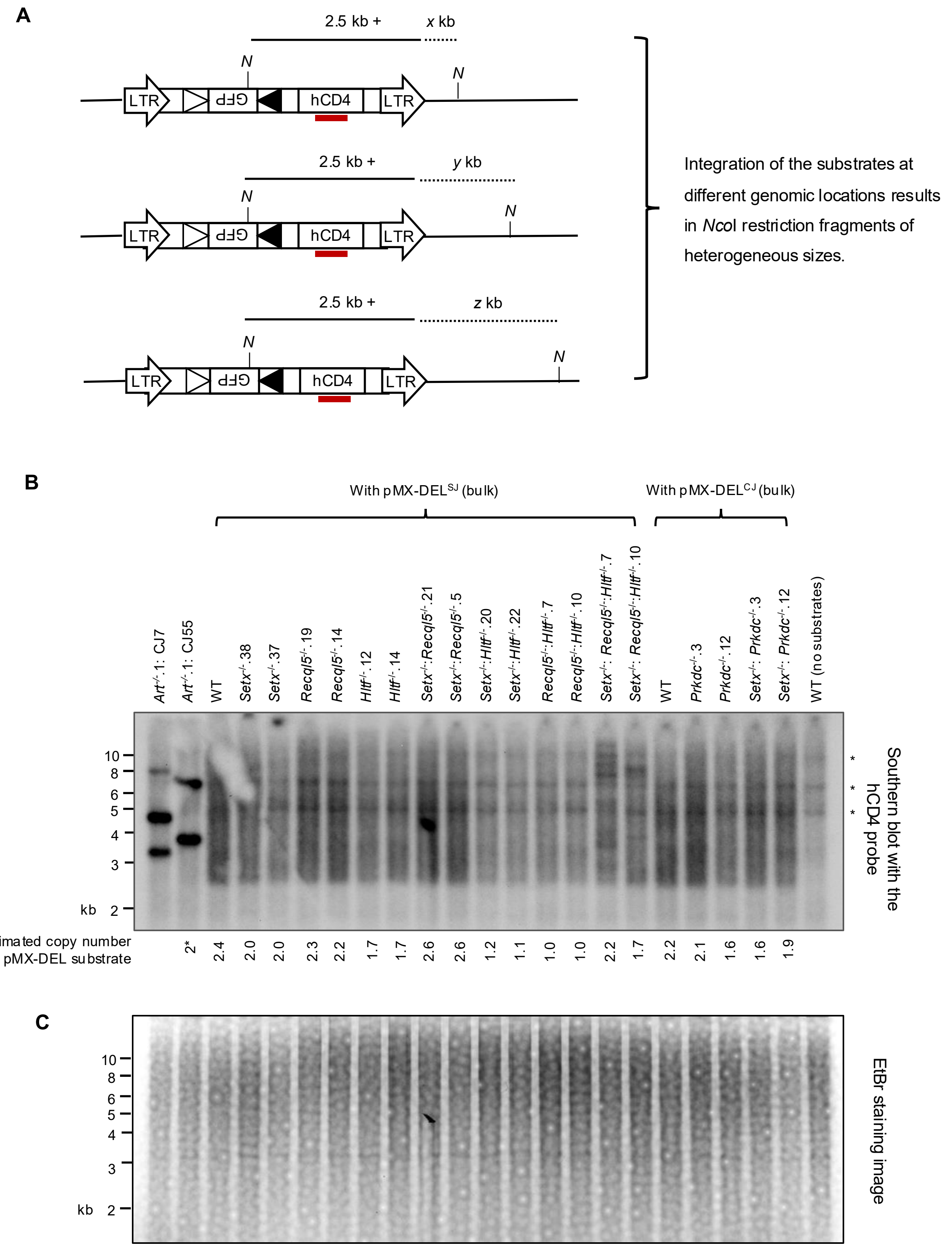

Figure S19: Analysis of the distribution and the estimated copy number of the pMX-DEL^{SJ} and pMX-DEL^{CJ} retroviral recombination substrates in bulk transduced abl-pre B cells. (A) The schematic of the generation of *Nco*I (N) restriction fragments heterogeneous in sizes upon the digestion of a unique *Nco*I site in the recombination substrate (pMX-DEL^{SJ} as an example) and genomic *NcoI* sites adjacent to intergradation sites in bulk transduced abl pre-B cells. (B) Southern blot analysis of genomic DNA from the indicated cell lines bulk transduced with pMX-DEL^{SJ} or pMX-DEL^{CJ} substrates. The genomic DNA samples were digested with *Nco*I and hybridized with the hCD4 probe (red bar in *(A)*). *Art*$^{-/-}$: CJ7 and *Art*$^{-/-}$: CJ55, clonal ARTEMIS-deficient abl pre-B cells with 3 and 2 copies of pMX-DEL^{CJ}, indicated by 3 and 2 distinct *Nco*I restriction fragments, were included as controls. The estimated copy numbers of the pMX-DEL substrates, determined by qPCR and normalized to *Art*$^{-/-}$: CJ55 (2* for 2 copies) using primers amplifying a unique region in the hCD4 cDNA on the recombination substrate, are shown in the bottom of the Southern blot image. * indicates non-specific hybridizing bands. (C) The EtBr image for *(B)* as the loading control.

Table S2. *Setx$^{-/-}$* abl pre-B cell line mutation sequencing

Cell line	Frequency	Sequence (resulting mutations)
WT	–	CCTTACGCCTCGTAAGTCACTTTGAGAAGTCCATGAAG
Setx$^{-/-}$.10	6/9	CCTTACCCCCCGTAAGTCACTTTGAGAAGTCCATGAAG (1)
	3/9	CCTTAC**^G^**GCCTCGTAAGTCACTTTGAGAAGTCCATGAAG (2)
Setx$^{-/-}$.37	5/12	CCTTAC**^G^**GCCTCGTAAGTCACTTTGAGAAGTCCATGAAG (2)
	7/12	CCTTACG**^GG^**CCTCGTAAGTCACTTTGAGAAGTCCATGAAG (3)
Setx$^{-/-}$.38	6/11	CCTTAC**^G^**GCCTCGTAAGTCACTTTGAGAAGTCCATGAAG (2)
	5/11	CCTTACG-CTCGTAAGTCACTTTGAGAAGTCCATGAAG (4)
*B.Setx$^{-/-}$.10**	10/10	CCTTAC**^G^**GCCTCGTAAGTCACTTTGAGAAGTCCATGAAG (2)
B.Setx$^{-/-}$.63	5/8	CCTTACCCCTCGTAAGTCACTTTGAGAAGTCCATGAAG (5) (R68P)
	3/8	CCTTACG**^GG^**CCTCGTAAGTCACTTTGAGAAGTCCATGAAG (3)
B.Setx$^{-/-}$.81	2/4	CCTTAC**^G^**GCCTCGTAAGTCACTTTGAGAAGTCCATGAAG (2)
	2/4	79 bp deletion(17 bp of intron 3 and 62 bp of exon 4 deleted)(6)

Resulting mutations:

(1) G to C and T to C mutations result in R68P and L69P in the amino acid sequence.
(2) ^G^: G insertion results in a premature stop codon
(3) ^GG^: GG insertion results in a premature stop codon
(4) – : C deletion results in a premature stop codon
(5) G to C mutation result in the missense mutation R68P.
(6) 79 bp deletion results in a premature stop codon.

* Only one mutated allele was detected. No WT allele was detected.

Table S3. Summary of GFP flow cytometric assays on pMG-INV recombination

For each mutant cell line, 3 independently isolated clones were analyzed. The average and standard deviation of the GFP+ cells are shown and calculated from 3 independent experiments . P values are determined by two-tailed Student's t test.

Table 3A

	Imatinib (days)				Imatinib + NU7441 (days)		
% GFP+	**0**	**2**	**3**	**4**	**2**	**3**	**4**
**WT**	1.6 ± 0.1	43.0 ± 10.3	71.2 ± 4.7	82.4 ± 4.9	35.7 ± 8.4	61.1 ± 7.3	71.4 ± 6.0
Setx$^{-/-}$**.38**	1.3 ± 0.9	25.4 ± 2.7	48.1 ± 2.2	61.9 ± 2.8	3.1 ± 1.7	6.2 ± 1.7	10.5 ± 2.3
Setx$^{-/-}$**.37**	2.2 ± 1.2	27.3 ± 5.2	50.6 ± 2.6	61.1 ± 1.7	3.3 ± 1.6	6.0 ± 1.9	8.6 ± 1.8
Setx$^{-/-}$**.10**	2.4 ± 1.6	20.3 ± 1.9	41.1 ± 2.2	52.1 ± 2.0	3.4 ± 2.1	6.8 ± 2.8	9.3 ± 2.5

	Imatinib (days)				Imatinib + NU7441 (days)		
P value*	**0**	**2**	**3**	**4**	**2**	**3**	**4**
**WT**	-	-	-	-	-	-	-
Setx$^{-/-}$**.38**	-	0.0812	0.0211	0.0212	0.0161	0.0044	0.0018
Setx$^{-/-}$**.37**	-	0.1074	0.0295	0.0245	0.0163	0.0039	0.0018
Setx$^{-/-}$**.10**	-	0.0516	0.0168	0.0141	0.0150	0.0035	0.0016

* *Setx*$^{-/-}$ vs WT at each time point for each treatment.

	Imatinib (days)				Imatinib + NU7441 (days)		
P value**	**0**	**2**	**3**	**4**	**2**	**3**	**4**
**WT**	-	-	-	-	0.2294	0.0807	0.0163
Setx$^{-/-}$**.38**	-	-	-	-	0.0014	0.0014	0.0001
Setx$^{-/-}$**.37**	-	-	-	-	0.0078	0.0003	0.0004
Setx$^{-/-}$**.10**	-	-	-	-	0.0013	0.0027	0.0011

** Imatinib + NU7441 vs imatinib only at each time point for each genotype.

Table S3B

	Imatinib (days)				Imatinib + KU55933 (days)		
% GFP+	0	2	3	4	2	3	4
**WT**	1.7 ± 0.2	43.1 ± 10.9	70.7 ± 9.5	82.4 ± 6.2	36.0 ± 4.1	59.4 ± 6.0	66.3 ± 5.4
Setx$^{-/-}$.38	2.5 ± 1.1	29.1 ± 3.6	50.3 ± 0.9	63.3 ± 1.6	25.0 ± 5.7	46.5 ± 1.0	56.8 ± 0.4
Setx$^{-/-}$.37	3.0 ± 1.3	34.0 ± 3.6	53.5 ± 4.7	63.1 ± 2.8	26.1 ± 2.8	47.4 ± 6.1	56.2 ± 5.6
Setx$^{-/-}$.10	3.2 ± 2.0	27.4 ± 3.6	46.5 ± 7.3	55.1 ± 4.4	24.6 ± 4.5	43.9 ± 8.7	51.3 ± 6.5

	Imatinib (days)				Imatinib + KU55933 (days)		
P value*	0	2	3	4	2	3	4
**WT**	-	-	-	-	-	-	-
***Setx$^{-/-}$*.38**	-	0.1909	0.0653	0.0365	0.1362	0.0845	0.0831
***Setx$^{-/-}$*.37**	-	0.3382	0.0996	0.0297	0.1112	0.0121	0.0021
***Setx$^{-/-}$*.10**	-	0.1558	0.0532	0.0146	0.1317	0.0228	0.0019

* *Setx$^{-/-}$* vs WT at each time point for each treatment.

	Imatinib (days)				Imatinib + KU55933 (days)		
P value**	0	2	3	4	2	3	4
**WT**	-	-	-	-	0.2116	0.1629	0.0531
Setx$^{-/-}$.38	-	-	-	-	0.0881	0.0081	0.0272
Setx$^{-/-}$.37	-	-	-	-	0.0129	0.0199	0.0530
Setx$^{-/-}$.10	-	-	-	-	0.2236	0.2776	0.1028

** Imatinib + KU55933 vs imatinib only at each time point for each genotype.

Table S3C

	Imatinib (days)				Imatinib + KU55933 (days)			Imatinib + NU7441 (days)		
% GFP+	**0**	**2**	**3**	**4**	**2**	**3**	**4**	**2**	**3**	**4**
**WT**	1.9 ± 0.3	34.9 ± 6.5	65.8 ± 6.0	79.5 ± 6.7	30.2 ± 7.7	54.8 ± 6.9	63.8 ± 5.9	31.6 ± 10.1	59.1 ± 8.8	69.5 ± 6.5
***Setx$^{-/-}$*.38**	1.8 ± 0.6	25.1 ± 2.8	49.3 ± 1.3	61.4 ± 2.2	23.7 ± 4.3	46.2 ± 2.4	55.7 ± 1.6	3.1 ± 0.8	6.9 ± 1.9	10.6 ± 3.3
***Recql5$^{-/-}$*.14**	1.7 ±1.6	43.6 ± 3.0	74.5 ± 1.1	85.6 ± 0.1	26.4 ± 3.4	50.7 ± 3.0	57.5 ± 2.4	37.3 ± 11.5	64.7 ± 8.0	74.5 ±5.4
***Recql5$^{-/-}$*.19**	3.8 ± 2.9	51.3 ± 2.3	79.6 ± 4.3	90.2 ± 3.5	30.7 ± 7.5	50.4 ± 6.7	56.0 ± 6.2	41.8 ± 13.9	66.7 ± 10.7	75.4 ±7.5
***Recql5$^{-/-}$*.35**	3.8 ±3.6	45.9 ± 5.7	72.0 ± 6.4	84.9 ± 5.3	25.5 ± 9.8	46.8 ± 12.3	53.2 ± 7.5	33.3 ± 15.8	60.0 ± 14.8	71.2 ±9.9

	Imatinib (days)				Imatinib + KU55933 (days)			Imatinib + NU7441 (days)		
P value*	**0**	**2**	**3**	**4**	**2**	**3**	**4**	**2**	**3**	**4**
**WT**	-	-	-	-	-	-	-	-	-	-
***Setx$^{-/-}$*.38**	-	0.1017	0.0281	0.0226	0.1782	0.0980	0.4960	0.0341	0.0061	0.0011
***Recql5$^{-/-}$*.14**	-	0.2361	0.1715	0.2565	0.3431	0.2204	0.1014	0.0188	0.0365	0.0561
***Recql5$^{-/-}$*.19**	-	0.2361	0.0116	0.0564	0.0669	0.0087	0.0015	0.0421	0.0220	0.0153
***Recql5$^{-/-}$*.35**	-	0.0212	0.0019	0.0445	0.0761	0.1217	0.0222	0.6821	0.7712	0.5499

* *Setx$^{-/-}$* or *Recql5$^{-/-}$* vs *WT* at each time point for each treatment.

Table S3D

	Imatinib (days)			
% GFP+	**0**	**2**	**3**	**4**
**WT**	1.6 ± 0.2	38.8 ± 0.7	66.6 ± 3.4	82.2 ± 2.1
***Setx$^{-/-}$*.38**	1.4 ± 0.6	30.5 ± 3.7	51.1 ± 1.0	64.3 ± 0.2
***Setx$^{-/-}$: Recql5$^{-/-}$*.5**	0.8 ± 0.2	23.7 ± 8.8	35.0 ± 8.8	42.7 ± 7.9
***Setx$^{-/-}$: Recql5$^{-/-}$*.21**	0.9 ± 0.2	22.9 ± 7.5	35.3 ± 8.5	43.4 ± 5.8
***Setx$^{-/-}$: Recql5$^{-/-}$*.45**	0.9 ± 0.4	22.7 ± 2.6	37.6 ± 6.9	44.7± 6.9

	Imatinib (days)			
P value*	**0**	**2**	**3**	**4**
**WT**	-	0.0603	0.0091	0.0042
***Setx$^{-/-}$*.38**	-			
***Setx$^{-/-}$: Recql5$^{-/-}$*.5**	-	0.2974	0.0705	0.0411
***Setx$^{-/-}$: Recql5$^{-/-}$*.21**	-	0.2608	0.0674	0.0244
***Setx$^{-/-}$: Recql5$^{-/-}$*.45**	-	0.1114	0.0595	0.0369

* WT or *Setx$^{-/-}$: Recql5$^{-/-}$* vs *Setx$^{-/-}$* at each time point.

Table S3E

	Imatinib (days)				Imatinib + KU55933 (days)			Imatinib + NU7441 (days)		
% GFP+	0	2	3	4	2	3	4	2	3	4
**WT**	2.1 ± 0.3	38.3 ± 8.1	66.3 ± 8.2	80.7 ± 6.8	34.9 ± 10.1	55.5 ± 6.1	64.6 ± 4.0	32.4 ± 4.9	59.3 ± 9.9	71.0 ± 7.1
Setx$^{-/-}$**.38**	1.8 ± 0.4	26.5 ± 1.2	48.9 ± 1.1	63.1 ± 1.2	26.4 ± 1.6	46.5 ± 2.6	57.4 ± 1.8	4.3 ± 0.2	7.3 ± 2.0	11.2 ± 2.7
Hltf$^{-/-}$**.12**	2.9 ± 2.0	41.0 ± 9.4	71.0 ± 6.0	86.2 ± 2.9	31.0 ± 3.8	55.2 ± 3.4	63.6 ± 1.8	41.8 ± 9.7	60.9 ± 9.1	72.8 ± 5.7
Hltf$^{-/-}$**.14**	2.1 ± 1.2	39.0 ± 7.9	72.9 ± 6.3	84.9 ± 5.6	35.1 ± 6.5	62.5 ± 4.0	69.5 ± 2.7	42.7 ± 13.4	71.8 ± 6.6	80.7 ± 4.1
Hltf$^{-/-}$**.16**	0.9 ± 0.5	32.0 ± 5.0	62.8 ± 7.1	80.6 ± 5.3	28.0 ± 6.2	53.7 ± 7.4	64.3 ± 4.8	32.9 ± 14.1	59.9 ± 15.6	73.0 ± 9.4

	Imatinib (days)				Imatinib + KU55933 (days)			Imatinib + NU7441 (days)		
P value*	0	2	3	4	2	3	4	2	3	4
**WT**	-	-	-	-	-	-	-	-	-	-
Setx$^{-/-}$**.38**	-	0.1123	0.0587	0.0322	0.2922	0.0660	0.0313	0.0091	0.0081	0.0020
Hltf$^{-/-}$**.12**	-	0.3317	0.2284	0.2251	0.3978	0.8706	0.5344	0.0833	0.6155	0.5429
Hltf$^{-/-}$**.14**	-	0.7692	0.0311	0.0448	0.9551	0.0384	0.0925	0.2387	0.0715	0.0705
Hltf$^{-/-}$**.16**	-	0.1818	0.3868	0.9655	0.1634	0.3756	0.6949	0.9399	0.9334	0.6150

* *Setx*$^{-/-}$ or *Hltf*$^{-/-}$ vs *WT* at each time point for each treatment.

Table S3F

	Imatinib (days)				Imatinib + KU55933 (days)			Imatinib + NU7441 (days)		
% GFP+	0	2	3	4	2	3	4	2	3	4
**WT**	1.7 ± 0.7	39.2 ± 2.5	68.9 ± 2.5	81.6 ± 2.9	36.2 ± 2.6	59.6 ± 3.1	65.7 ± 3.0	39.1 ± 8.2	63.1 ± 5.5	73.1 ± 4.9
***Setx*$^{-/-}$.38**	1.8 ± 0.3	27.6 ± 2.5	51.9 ± 1.8	65.3 ±1.5	30.9 ± 5.2	51.4 ± 3.4	59.9 ± 1.0	4.3 ± 1.3	8.3 ± 0.9	11.8 ± 1.2
***Setx*$^{-/-}$: *Hltf*$^{-/-}$.13**	0.7 ± 0.2	30.1 ± 5.3	55.2 ± 2.5	68.7 ± 0.5	27.8 ± 3.0	52.4 ± 2.3	62.0 ± 0.8	3.2 ± 0.9	7.9 ± 2.3	12.0 ± 3.6
***Setx*$^{-/-}$: *Hltf*$^{-/-}$.20**	0.5 ± 0.2	27.2 ± 2.9	52.8 ± 2.5	64.4 ± 1.7	30.5 ± 0.2	52.3 ± 1.0	58.2 ± 1.2	2.3 ± 0.6	5.2 ± 1.0	8.3 ± 2.5
***Setx*$^{-/-}$: *Hltf*$^{-/-}$.22**	0.7 ± 0.5	25.1 ± 4.4	51.6 ± 3.6	66.6 ± 3.3	28.4 ± 0.8	53.5 ± 0.8	62.4 ± 1.1	2.6 ± 0.6	6.4 ± 1.3	10.0 ± 1.7

	Imatinib (days)				Imatinib + KU55933 (days)			Imatinib + NU7441 (days)		
P value*	0	2	3	4	2	3	4	2	3	4
**WT**	-	0.0180	0.0218	0.0384	0.1555	0.1173	0.0521	0.0102	0.0025	0.0015
***Setx*$^{-/-}$.38**	-									
***Setx*$^{-/-}$: *Hltf*$^{-/-}$.13**	-	0.9745	0.9029	0.7352	0.4327	0.5545	0.8612	0.3014	0.0282	0.1120
***Setx*$^{-/-}$: *Hltf*$^{-/-}$.20**	-	0.6034	0.7344	0.2972	0.8035	0.8123	0.3311	0.0965	0.0656	0.0649
***Setx*$^{-/-}$: *Hltf*$^{-/-}$.22**	-	0.3843	0.5194	0.6515	0.4438	0.5962	0.6425	0.1769	0.0024	0.0332

* WT or *Setx*$^{-/-}$:*Hltf*$^{-/-}$ vs *Setx*$^{-/-}$ at each time point for each treatment.

Table S3G

	Imatinib (days)				Imatinib + KU55933 (days)		
% GFP+	0	2	3	4	2	3	4
**WT**	1.6 ± 0.2	41.8 ± 2.5	68.7 ± 2.7	82.7 ± 1.9	31.9 ± 2.0	55.4 ± 2.5	63.1 ± 3.4
***Setx*$^{-/-}$.38**	1.5 ± 0.3	28.4 ± 3.2	50.3 ± 0.6	63.8 ± 1.2	24.9 ± 4.0	47.7 ± 1.8	58.5 ± 1.9
***Recql5*$^{-/-}$: *Hltf*$^{-/-}$.7**	3.9 ± 1.2	51.3 ± 0.7	77.2 ± 1.2	87.9 ± 0.5	17.3 ± 0.9	31.0 ± 2.6	37.3 ± 2.1
***Recql5*$^{-/-}$: *Hltf*$^{-/-}$.10**	4.2 ± 0.9	39.6 ± 4.3	67.1 ± 3.2	82.7 ± 3.5	10.8 ± 1.3	21.7 ± 1.8	28.3 ± 3.0
***Recql5*$^{-/-}$: *Hltf*$^{-/-}$.15**	3.4 ± 0.8	45.2 ± 5.6	71.5 ± 4.1	85.2 ± 2.7	17.6 ± 1.2	32.4 ± 1.1	38.1 ± 1.5

	Imatinib (days)				Imatinib + KU55933 (days)		
P value*	0	2	3	4	2	3	4
**WT**	-	0.0418	0.0088	0.0086	0.0269	0.0259	0.0363
***Setx*$^{-/-}$.38**	-	-	-	-	-	-	-
***Recql5*$^{-/-}$: *Hltf*$^{-/-}$.7**	-	0.0050	0.0005	0.0013	0.0620	0.0029	0.0016
***Recql5*$^{-/-}$: *Hltf*$^{-/-}$.10**	-	0.0036	0.0084	0.0159	0.0120	0.0019	0.0057
***Recql5*$^{-/-}$: *Hltf*$^{-/-}$.15**	-	0.0166	0.0095	0.0068	0.0641	0.0009	0.0038

* WT or *Recql5*$^{-/-}$:*Hltf*$^{-/-}$ vs *Setx*$^{-/-}$ at each time point for each treatment.

	Imatinib (days)				Imatinib + KU55933 (days)		
P value**	0	2	3	4	2	3	4
**WT**	-	-	-	-	-	-	-
***Setx*$^{-/-}$.38**	-	0.0418	0.0088	0.0086	0.0269	0.0259	0.0363
***Recql5*$^{-/-}$: *Hltf*$^{-/-}$.7**	-	0.0194	0.0261	0.0456	0.0044	0.0078	0.0029
***Recql5*$^{-/-}$: *Hltf*$^{-/-}$.10**	-	0.5863	0.6023	0.9902	0.0003	0.0042	0.0079
***Recql5*$^{-/-}$: *Hltf*$^{-/-}$.15**	-	0.4030	0.4387	0.3751	0.0030	0.0038	0.0071

** Mutants vs WT at each time point for each treatment.

	Imatinib (days)				Imatinib + KU55933 (days)		
P value***	0	2	3	4	2	3	4
**WT**	-	-	-	-	0.0597	0.0058	0.0033
***Setx*$^{-/-}$.38**	-	-	-	-	0.1437	0.1802	0.0712
***Recql5*$^{-/-}$: *Hltf*$^{-/-}$.7**	-	-	-	-	0.0007	0.0021	0.0011
***Recql5*$^{-/-}$: *Hltf*$^{-/-}$.10**	-	-	-	-	0.0040	0.0022	0.0034
***Recql5*$^{-/-}$: *Hltf*$^{-/-}$.15**	-	-	-	-	0.0094	0.0055	0.0021

*** Imatinib + KU55933 vs imatinib only for each cell line at each time point .

Table S3H

	Imatinib (days)			
% GFP+	**0**	**2**	**3**	**4**
**WT**	1.8 ± 0.1	39.6 ± 1.2	67.6 ± 2.4	81.2 ± 2.4
***Setx$^{-/-}$*.38**	1.4 ± 0.7	29.0 ± 2.2	51.2 ± 1.7	64.3 ± 1.4
***Setx$^{-/-}$:Recql5$^{-/-}$: Hltf$^{-/-}$*.36**	0.4 ± 0.1	10.3 ± 2.3	18.4 ± 3.9	24.2 ± 5.0
***Setx$^{-/-}$:Recql5$^{-/-}$: Hltf$^{-/-}$*.38**	0.5 ± 0.2	9.3 ± 1.6	17.5 ± 2.5	24.4 ±3.5
***Setx$^{-/-}$:Recql5$^{-/-}$: Hltf$^{-/-}$*.39**	0.7 ± 0.2	11.7 ± 1.1	21.3 ± 2.5	27.1 ± 2.9

	Imatinib (days)			
P value*	**0**	**2**	**3**	**4**
**WT**	-	0.0298	0.0102	0.0066
***Setx$^{-/-}$*.38**	-	-	-	-
***Setx$^{-/-}$:Recql5$^{-/-}$: Hltf$^{-/-}$*.38**	-	0.0142	0.0102	0.0041
***Setx$^{-/-}$:Recql5$^{-/-}$: Hltf$^{-/-}$*.39**	-	0.0013	0.0008	0.0011
***Setx$^{-/-}$:Recql5$^{-/-}$: Hltf$^{-/-}$*.36**	-	0.0427	0.0027	0.0012

* WT or *Setx$^{-/-}$: Recql5$^{-/-}$: Hltf$^{-/-}$* vs *Setx$^{-/-}$* at each time point.

Table S3I

% GFP+	Imatinib (days)				Imatinib + NU7441 (days)		
	0	2	3	4	2	3	4
**WT**	1.9 ± 0.1	42.5 ± 6.5	71.4 ± 4.1	83.4 ± 2.5	40.1 ± 6.2	63.6 ± 7.3	73.1 ± 4.7
***Setx$^{-/-}$*.38**	2.1 ± 0.3	30.1 ± 2.8	51.9 ± 3.6	64.5 ± 2.9	4.2 ± 1.5	8.3 ± 2.7	12.8 ± 2.8
***Recql5$^{-/-}$*: *Hltf$^{-/-}$*.7**	4.6 ± 1.2	47.3 ± 4.2	75.5 ± 2.5	87.3 ± 2.2	47.3 ± 7.1	69.8 ± 5.3	77.8 ± 3.9
***Recql5$^{-/-}$*: *Hltf$^{-/-}$*.10**	4.5 ± 0.7	34.8 ± 8.3	65.9 ± 7.0	82.9 ± 4.4	38.1 ± 0.6	64.7 ± 3.0	74.6 ± 2.9
***Recql5$^{-/-}$*: *Hltf$^{-/-}$*.15**	4.5 ± 0.9	45.8 ± 3.6	73.0 ± 2.1	86.6 ± 0.8	39.1 ± 4.0	63.5 ± 4.7	74.8 ± 3.1

P value*	Imatinib (days)				Imatinib + NU7441 (days)		
	0	2	3	4	2	3	4
**WT**	-	-	-	-	-	-	-
***Setx$^{-/-}$*.38**	0.1392	0.0316	0.0174	0.0087	0.0044	0.0019	0.1392
***Recql5$^{-/-}$*: *Hltf$^{-/-}$*.7**	0.3936	0.3302	0.1158	0.1098	0.1098	0.0895	0.3936
***Recql5$^{-/-}$*: *Hltf$^{-/-}$*.10**	0.1756	0.3318	0.8471	0.6499	0.7042	0.3169	0.1756
***Recql5$^{-/-}$*: *Hltf$^{-/-}$*.15**	0.5032	0.6819	0.1654	0.6691	0.9846	0.4325	0.5032

*Mutants vs WT at each time point for each treatment.

P value**	Imatinib (days)				Imatinib + NU7441 (days)		
	0	2	3	4	2	3	4
**WT**	-	-	-	-	0.6207	0.0601	0.0176
***Setx$^{-/-}$*.38**	-	-	-	-	0.0040	0.0007	0.0002
***Recql5$^{-/-}$*: *Hltf$^{-/-}$*.7**	-	-	-	-	0.9963	0.3285	0.0827
***Recql5$^{-/-}$*: *Hltf$^{-/-}$*.10**	-	-	-	-	0.5395	0.7924	0.0856
***Recql5$^{-/-}$*: *Hltf$^{-/-}$*.15**	-	-	-	-	0.2628	0.1346	0.0202

** Imatinib + NU7441 vs imatinib only for each cell line at each time point

Table S3J

	Imatinib (days)				Imatinib + NU7441 (days)		
% GFP+	**0**	**2**	**3**	**4**	**2**	**3**	**4**
**WT**	0.5 ± 0.2	39.5 ± 1.1	67.5 ± 0.9	77.6 ± 1.1	36.2 ± 4.0	61.9 ± 3.9	71.3 ± 3.6
Setx$^{-/-}$:Recql5$^{-/-}$: Hltf$^{-/-}$.36	0.5 ± 0.3	6.9 ± 1.6	17.9 ± 3.4	25.1 ± 4.2	0.8 ± 0.4	2.9 ± 0.8	4.2 ± 1.2
Setx$^{-/-}$:Recql5$^{-/-}$: Hltf$^{-/-}$.38	0.5 ± 0.2	8.2 ± 3.0	19.6 ± 4.0	25.4 ± 6.5	0.9 ± 0.3	2.4 ± 0.7	4.8 ± 0.5
Setx$^{-/-}$:Recql5$^{-/-}$: Hltf$^{-/-}$.39	0.2 ± 0.1	7.2 ± 1.4	18.9 ± 3.0	27.6 ± 5.2	0.8 ± 0.3	2.7 ± 0.6	5.3 ± 0.7

	Imatinib (days)				Imatinib + NU7441 (days)		
P value*	**0**	**2**	**3**	**4**	**2**	**3**	**4**
**WT**	-	-	-	-	0.2735	0.1363	0.1393
Setx$^{-/-}$:Recql5$^{-/-}$: Hltf$^{-/-}$.36	-	-	-	-	0.0146	0.0242	0.0076
Setx$^{-/-}$:Recql5$^{-/-}$: Hltf$^{-/-}$.38	-	-	-	-	0.0419	0.0127	0.0305
Setx$^{-/-}$:Recql5$^{-/-}$: Hltf$^{-/-}$.39	-	-	-	-	0.0110	0.0081	0.0178

* Imatinib + NU7441 vs imatinib only at each time point for each genotype.

Table S8. Oligo nucleotides sequences.

Name	Sequence	Note
Primers for CRISPR/Cas9 gRNA screen sequencing		
pKLV lib330F:	AATGGACTATCATATGCTTACCGT	For gRNA library sequencing (1st round PCR)
pKLV lib490R:	CCTACCGGTGGATGTGGAATG	For gRNA library sequencing (1st round PCR)
PE.P5_pKLV lib195 Fwd	**AATGATACGGCGACCACCGAGATCTACAC**GGCTTTATATATCTTGTGGAAAGGAC	For gRNA library sequencing (**P5 adaptor**)
P7 index180 Rev:	**CAAGCAGAAGACGGCATACGAGAT**-index-*GTGACTGGAGTTCAGACGTGTGCTCTTCCGATC*CAGACTGCCTTGGGAAAAGC	For gRNA library sequencing (**P7 adaptor,** *Illumina sequencing primer*)
gRNA library seq Read 1 primer	GGCTTTATATATCTTGTGGAAAGGACGAAACACCG	
gRNAs for gene inactivation		
gSetx	AAGTGACTTACGAGGCGTA	
gRecql5	CGTTGCAGCTCGACTCAGG	
gHltf	TCTGTACTGCCACATGAGC	
gPrkdc	ATGCGTCTTAGGTGATCGA	
gEb	GAAAGCCAGCCAATGAATGC	
Primers for SJ PCR and sequencing		
pMG-INV SJ F4	CGGCATCAAGGCGAACTTCA	For SJ sequencing (1st round PCR)
pMG-INV 3'-3 R	GTAAAGCATGTGCACCGAGG	For SJ sequencing (1st round PCR)
PE.P5_SJ23	**AATGATACGGCGACCACCGAGATCTACAC**CTGACTTGAATCATGTTGTTTTCCAGACTT	For SJ sequencing (**P5 adaptor**)
P7_SJ12	**CAAGCAGAAGACGGCATACGAGAT-**index-*GTGACTGGAGTTCAGACGTGTGCTCTTCCGATC*CCAAGCGGCTTCGGCCAGTAACGTT	For SJ sequencing (**P7 adaptor,** *Illumina sequencing primer*)
SJ seq Read 1 primer	CTGACTTGAATCATGTTGTTTTCCAGACTTCAACT	

Data S1_SJ seq reference

```
LOCUS       Signal_join_miSe         295 bp DNA     linear   UNA 30-JUN-
2024
DEFINITION  natural linear DNA
ACCESSION   .
VERSION     .
KEYWORDS    .
SOURCE      natural DNA sequence
  ORGANISM  unspecified
REFERENCE   1  (bases 1 to 295)
  AUTHORS   .
  TITLE     Direct Submission
  JOURNAL   Exported Jun 30, 2024 from SnapGene Viewer 6.2.2
            https://www.snapgene.com
FEATURES             Location/Qualifiers
     source          1..295
                     /mol_type="genomic DNA"
                     /organism="unspecified"
     primer_bind     1..30
                     /label=(Partial) PE.P5_SJ23
     iDNA            complement(127..165)
                     /label=23 RSS
     misc_feature    166..193
                     /label=12 RSS
     primer_bind     complement(271..295)
                     /label=(Partial) P7_SJ12
ORIGIN
1     ctgacttgaa tcatgttgtt ttccagactt caacttgact atcagccaga aattcagtgg
61    caaacccccct ccacccatcc ctagtgaggg ttcctagtga gggaggaagg actaactcga
121   gggtgtggtt tttgtacagc cagacagtgg agtactacca ctgtgcacag tgctacagac
181   tggaacaaaa acagaccctc gttggccgcc accgatctct cgaggtcgac ggtatcgata
241   agcttgatat cgaattccgc ccccccccct aacgttactg gccgaagccg cttgg
//
```

Supplemental Excel files (uploaded as separate files)**:**

Table S1. The CRISPR/Cas9 gRNA screen result for genes required for V(D)J recombination in WT abl pre-B cells treated with the DNA-PKcs inhibitor NU7441.

Table S4. The CRISPR/Cas9 gRNA screen result for genes required for V(D)J recombination in *Setx*$^{-/-}$.38 abl pre-B cells.

Table S5. The CRISPR/Cas9 gRNA screen result for genes required for V(D)J recombination in *B.Setx*$^{-/-}$.10 abl pre-B cells.

Table S6. The CRISPR/Cas9 gRNA screen result for genes required for V(D)J recombination in *Setx*$^{-/-}$: *Recql5*$^{-/-}$.5 abl pre-B cells.

Table S7. Sequence analysis of SJs from pMG-INV from indicated abl pre-B cells.
